# Circular Dichroism and Multiphoton Circularly Polarized Luminescence Switching Using a Bis‐perylene Diimide Macrocycle

**DOI:** 10.1002/chem.202501734

**Published:** 2025-08-22

**Authors:** Samuel E. Penty, Martin V. Appleby, Martijn A. Zwijnenburg, Dominic J. Black, Denis Hartmann, Dimitri Chekulaev, Julia Weinstein, Robert Pal, Timothy A. Barendt

**Affiliations:** ^1^ School of Chemistry University of Birmingham Edgbaston Birmingham B15 2TT UK; ^2^ Department of Chemistry University of Durham South Road Durham DH1 3LE UK; ^3^ School of Mathematical and Physical Sciences University of Sheffield Dainton Building, 13 Brook Hill Sheffield S3 7HF UK; ^4^ Department of Chemistry University College London 20 Gordon Street London WC1H 0AJ UK

**Keywords:** chiral switch, circularly polarized luminescence, macrocycle, perylene diimide, supramolecular chirality

## Abstract

Chiroptical switches exhibit stimuli‐induced changes in the sign or strength of their circular dichroism (CD) or circularly polarized luminescence (CPL) and are materials with significant potential for chiroptical sensing and biological imaging. However, the chromophores used in chiroptical materials usually require excitation by ultraviolet or visible light, which results in higher cytotoxicity and lower penetration compared to near‐infrared (NIR) photoexcitation. This can be overcome by multiphoton excitation, yet multiphoton CPL (MP‐CPL) has been challenging to measure in organic emitters. Core‐twisted perylene diimides (PDIs) are excellent chromophores with which to develop MP‐CPL emitters due to a chiral π‐system capable of two‐photon absorption. Furthermore, the integration of these chiral PDIs into stimuli‐responsive macrocycles affords configurational stability and the potential for chiroptical switching, due to the chromophore's tuneable chiral conformation. In this work we report a chiral bis‐PDI macrocycle with stable, yet conformationally dynamic, enantiomers, and in doing so realize a novel chiroptical material that combines switchable CD and MP‐CPL for the first time.

## Graphical abstract

A chiral and configurationally stable bis‐perylene diimide macrocycle is reported, which exhibits switchable circular dichroism (CD) and multiphoton circularly polarized luminescence (MP‐CPL). This behavior is realized due to the macrocycle's tuneable chiral conformation, which impacts the intrinsic and supramolecular helicity of the perylene diimide dimer.

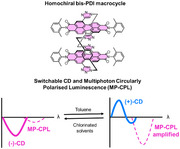

## 1 Introduction

From single molecules to supramolecular assemblies, organic dye‐based materials that absorb or emit circularly polarized light^[^ ^1^, ^2^, ^3^, ^4^ ^]^ have huge potential in applications such as circularly polarized OLEDs,^[^ ^5^, ^6^, ^7^ ^]^ security inks,^[^ ^8^, ^9^ ^]^ information transfer,^[^ ^10^ ^]^ and biological imaging and sensing.^[^ ^11^ ^]^ Critical to advancing chiroptical performance, the analysis of these materials using circular dichroism (CD) and circularly polarized luminescence (CPL) spectroscopy provides fundamental knowledge of the chirality of the chromophore's respective ground and excited states. These efforts are also important in the context of chiroptical switches,^[^ ^12^ ^]^ where a stimulus‐induced change in the CD/CPL signal sign (+/−) or amplitude has significant potential in the fields of chemical sensing and bioimaging.^[^ ^13^, ^14^, ^15^ ^]^ Here, molecular chiroptical switches are required to be triggered by chiral or achiral stimuli. The latter is arguably more challenging for +/− switching,^[^ ^16^, ^17^, ^18^, ^19^, ^20^, ^21^, ^22^ ^]^ since it requires the chromophore to exhibit both dynamic chirality and configurational stability, which is not the case for many contorted aromatic dyes.^[^ ^23^, ^24^, ^25^, ^26^ ^]^ Instead, dynamic chirality often goes in hand with a susceptibility to racemization, making the induction of chiroptical properties only possible with chiral stimuli.^[^ ^27^, ^28^, ^29^, ^30^ ^]^ Alternatively, the chromophore's rigidity may prevent the population of distinct chiral conformations and so precludes chiroptical switching altogether.^[^ ^31^, ^32^, ^33^, ^34^ ^]^

The chromophores used in chiroptical materials typically require excitation by ultraviolet or visible light^[^ ^35^, ^36^ ^]^ which, for biological applications, can result in higher cytotoxicity and lower penetration compared to near‐infrared (NIR) photoexcitation. Multiphoton (MP) spectroscopy uses a highly focused low‐energy near infrared (NIR) laser pulse to stimulate emission from luminescent molecules that would otherwise require absorption of a single high‐energy UV/visible photon.^[^ ^37^, ^38^ ^]^ As such, MP excitation is highly advantageous for bioimaging. In a similar fashion, multiphoton excitation CPL (MP‐CPL) would be advantageous for CPL‐based sensing and imaging in biological contexts.^[^ ^11^, ^39^ ^]^ However, the field of MP‐CPL is largely unexplored,^[^ ^11^, ^39^, ^40^, ^41^ ^]^ and, to the best of our knowledge, switchable MP‐CPL has not been reported from an organic emitter.

**Figure 1:**
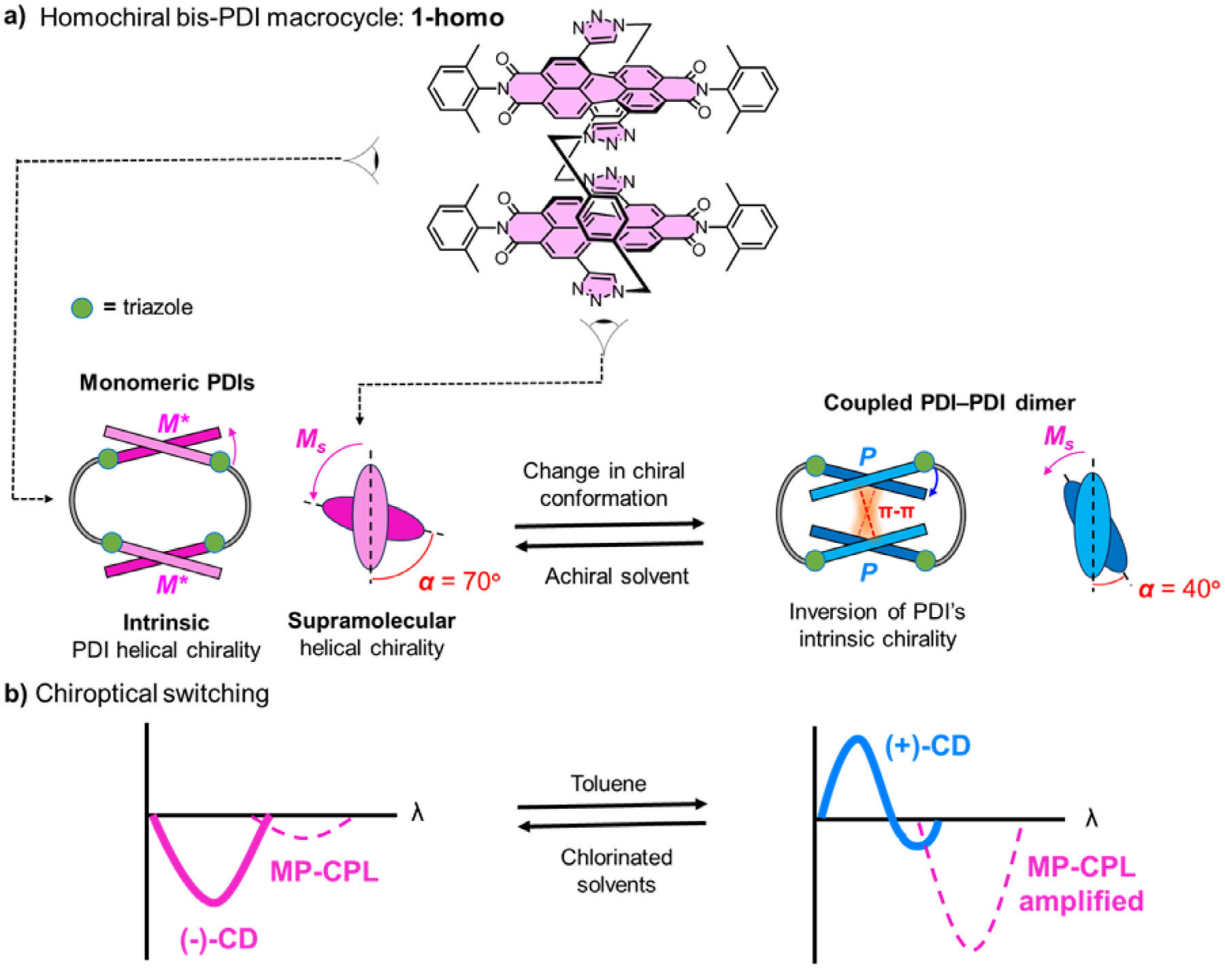
An outline of this work showing, **a)** the achiral stimulus‐induced change in chiral conformation within bis‐PDI macrocycle **1‐homo** and, **b)** the impact of this change on switching the sign and amplitude of **1‐homo**’s CD and MP‐CPL spectra, respectively. For the PDI's intrinsic helicity, M* indicates the pseudo‐enantiomeric relationship to ***P***.

Perylene diimides (PDIs) are a class of organic dyes with readily tunable photophysical, electrochemical, and supramolecular properties.^[^ ^42^ ^]^ Important for the design of multiphoton‐active chiroptical materials, some PDI dyes have been shown to exhibit excellent two‐photon absorption cross‐sections,^[^ ^43^, ^44^ ^]^ while others boast strong chiroptical properties.^[^ ^45^, ^46^ ^]^ These chiroptical properties may arise from the helical arrangement of two or more PDI monomers, i.e., supramolecular helicity (***M_s_***/***P_s_***, Figure 1a),^[^ ^47^ ^]^ which gives rise to excitonic chirality if there is excitonic coupling between the chromophores.^[^ ^48^, ^49^, ^50^, ^51^, ^52^, ^53^ ^]^ The PDI monomer can also be intrinsically chiral if functionalization of the PDI's bay positions (C1,6,7,12) leads to helical twisting of the perylene core (***M***/***P***, Figure 1a).^[^ ^54^, ^55^ ^]^ However, the majority of core‐twisted PDIs are not configurationally stable and so their chiroptical signal is not persistent,^[^ ^54^, ^55^ ^]^ limiting their value for fundamental analysis and the switching of chiroptical properties by achiral stimuli. In contrast to systems exploiting supramolecular helicity,^[^ ^47^, ^56^ ^]^ the PDI's intrinsic (molecular) helicity has only been used for solvent‐induced on/off switching of the CD/CPL signal.^[^ ^57^ ^]^ Core‐twisted PDIs have yet to be exploited for +/− chiroptical switching, which requires a configurationally stable and conformationally flexible PDI‐based architecture.

Configurationally stable core‐twisted PDIs may be realized by their integration into macrocycles, via the strapping of their bay positions.^[^ ^58^, ^59^, ^60^ ^]^ Bay‐connected bis‐PDI macrocycles that prevent the intramolecular somersault of the PDI units through the macrocycle's cavity may afford the resolution of homochiral enantiomers (***MM***, ***PP***) and the meso isomer (***MP***),^[^ ^46^, ^61^ ^]^ although it should be noted that a configurationally stable example of the latter has yet to be isolated. Furthermore, these chiral macrocycles provide a unique opportunity to explore the relationship between the core‐twisted PDI's intrinsic helicity (***M***/***P***) and the supramolecular helicity (***M_s_***/***P_s_***) of the PDI dimer (Figure 1a).

In this work, we report a bis‐PDI macrocycle (**1**) that, through a judicious choice of imide substituents, enables us to isolate and investigate the (chir)optical properties of configurationally stable homochiral enantiomers (**1‐homo**) and the meso isomer (**1‐meso**). Distinct from our previous bis‐PDI macrocycles,^[^ ^45^, ^46^ ^]^ the PDI units in **1** cannot undergo a somersaulting motion but can still invert their ***M***/***P*** core‐twist, which impacts intramolecular exitonic coupling in the PDI dimer (Figure 1a). Therefore, macrocycle **1** affords chiroptical switching by combining configurational stability and conformational flexibility. Specifically, we report (achiral) solvent‐induced switching of the sign and amplitude of **1‐homo's** CD and CPL spectra, respectively (Figure 1b), unique behavior for intrinsically chiral (core‐twisted) PDIs. Furthermore, since macrocycle **1** is multiphoton active, we record MP‐CPL spectra and so demonstrate switchable MP‐CPL for the first time in an organic emitter. By connecting the macrocycle's CD and (MP)‐CPL properties to changes in its chiral conformation, we identify the different roles of intrinsic and supramolecular helicity in defining the chiroptical response, important knowledge for the design of functional chiroptical switches.

### 1.1 Synthesis and Characterisation

**Figure 2:**
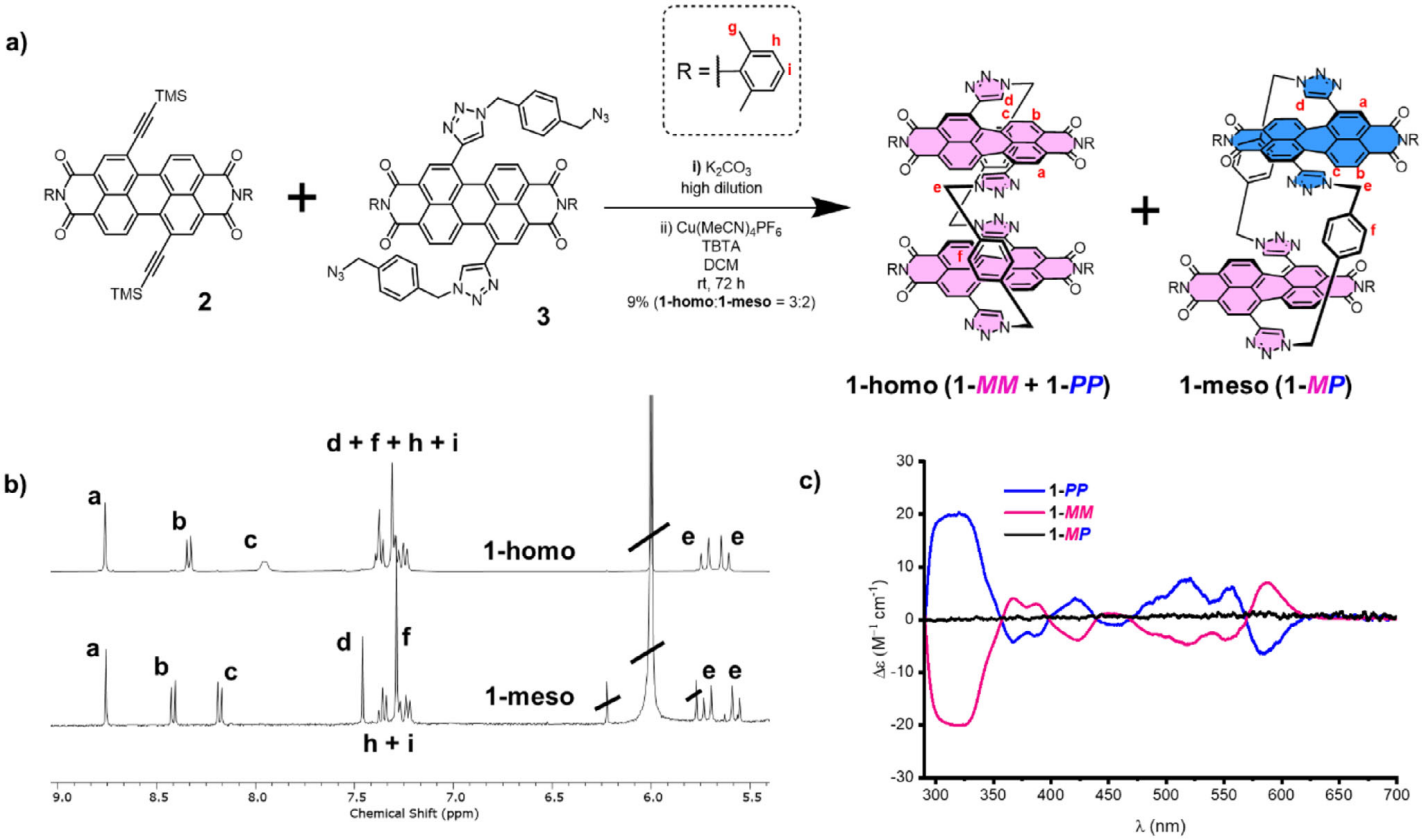
**a)** Synthesis of the macrocycle stereoisomers **1‐homo** and **1‐meso**. **b)** ^1^H NMR spectra (TCE‐*d*_2_, 343 K, 400 MHz) of **1‐homo** and **1‐meso**. **c)** CD spectra of **1‐homo** (**1‐*PP*** and **1‐*MM***) and **1‐meso** (**1‐*MP***) (10 µM, toluene).

For two reasons, we chose to functionalize the PDIs of the target macrocycle with 2,6‐dimethyl phenyl groups at the imide positions (Figure 2a). Firstly, the rigid aryl ring elongates the chromophore to ensure configurational stability.^[^ ^46^ ^]^ Secondly, the 2,6‐dimethyl groups may disrupt templating π–π stacking interactions^[^ ^45^ ^]^ during the macrocycle synthesis to enable formation of the meso isomer (***MP***) as well as the homochiral enantiomers (***MM***, ***PP***).

Following installation of the new 2,6‐dimethyl phenyl imide groups, the bis‐PDI macrocycle precursors bis‐alkyne **2** and bis‐azide **3**, were prepared according to the synthetic procedures outlined in the Supporting Information (Section 2). From here, macrocycle **1** was synthesised by a Cu(I) catalysed azide‐alkyne cycloaddition (CuAAC) ring‐closing strategy in which **2** is reacted with **3** in dichloromethane under high dilution conditions (Figure 2a). Preparatory scale high performance liquid chromatography (HPLC) was used to isolate two closely eluting compounds, which both had high‐resolution mass spectra consistent with the target bis‐PDI macrocycle **1** (Figure S3‐1). These compounds had similar, yet distinct, ^1^H NMR spectra (Figure 2b), suggesting the isolation of the homochiral enantiomers and the meso isomer in a 3:2 ratio, bis‐PDI macrocycles **1‐homo** (**1**‐***MM*** and **1**‐***PP***) and **1‐meso** (**1**‐***MP***), respectively (Figure 2a). Indeed, this outcome was confirmed by CD spectroscopy (Figure 2c). In contrast to our previous bis‐PDI macrocycle design,^[^ ^45^ ^]^ the bulky imide substituents of **1** are successful in hindering homochiral π–π templation during macrocyclization, thereby allowing us to isolate a configurationally stable meso isomer of a bis‐PDI macrocycle^[^ ^62^ ^]^ (**1‐meso**) which, for the first time, is solely connected through the bay positions.

To confirm the identity of the stereoisomers of **1**, both compounds were analysed by chiral HPLC and CD spectroscopy. As expected, **1‐homo** was resolved into its two enantiomers **1**‐***MM*** and **1**‐***PP*** (Figure S3‐2). The other compound is **1‐meso** (i.e., **1**‐***MP***) and so gave only a single peak (Figure S3‐3), consistent with it being a meso isomer. While **1‐meso** is achiral and hence CD silent, **1**‐***MM*** and **1**‐***PP*** have equal and opposite CD spectra in toluene (Figure 2c). The enantiomers are readily assigned using the CD spectra of **1**‐***PP*** and **1**‐***MM*** predicted by time‐dependent density functional theory (TD‐DFT) calculations in toluene (Supporting Information Section 8) as well as by comparison with the CD spectra of related bis‐PDI macrocycles.^[^ ^45^, ^46^, ^63^ ^]^

Compared to flexible branched alkyl chains,^[^ ^46^ ^]^ the bulky 2,6‐dimethyl phenyl imide groups prevent an intramolecular somersault of the PDI units and so ensure that macrocycle **1** is configurationally stable (Δ*G* ^‡^ > 155 kJ mol^−1^). This was evidenced by unchanged chiral HPLC chromatograms after heating **1‐meso** and the enantiomers of **1‐homo** for 24 hours at 180 °C in 1,2‐dichlorobenzene (Figure S3‐2 and S3‐3). This is important since, in bay‐connected bis‐PDI macrocycles that are not chirally locked, the meso isomer is a transient species (t_1/2_ is on the order of minutes) formed during the interconversion between enantiomers by an intramolecular somersault,^[^ ^46^, ^61^ ^]^ which, until now, has prevented its isolation and analysis.

### 1.2 Conformation‐dependent Photophysics of 1‐homo

**Figure 3:**
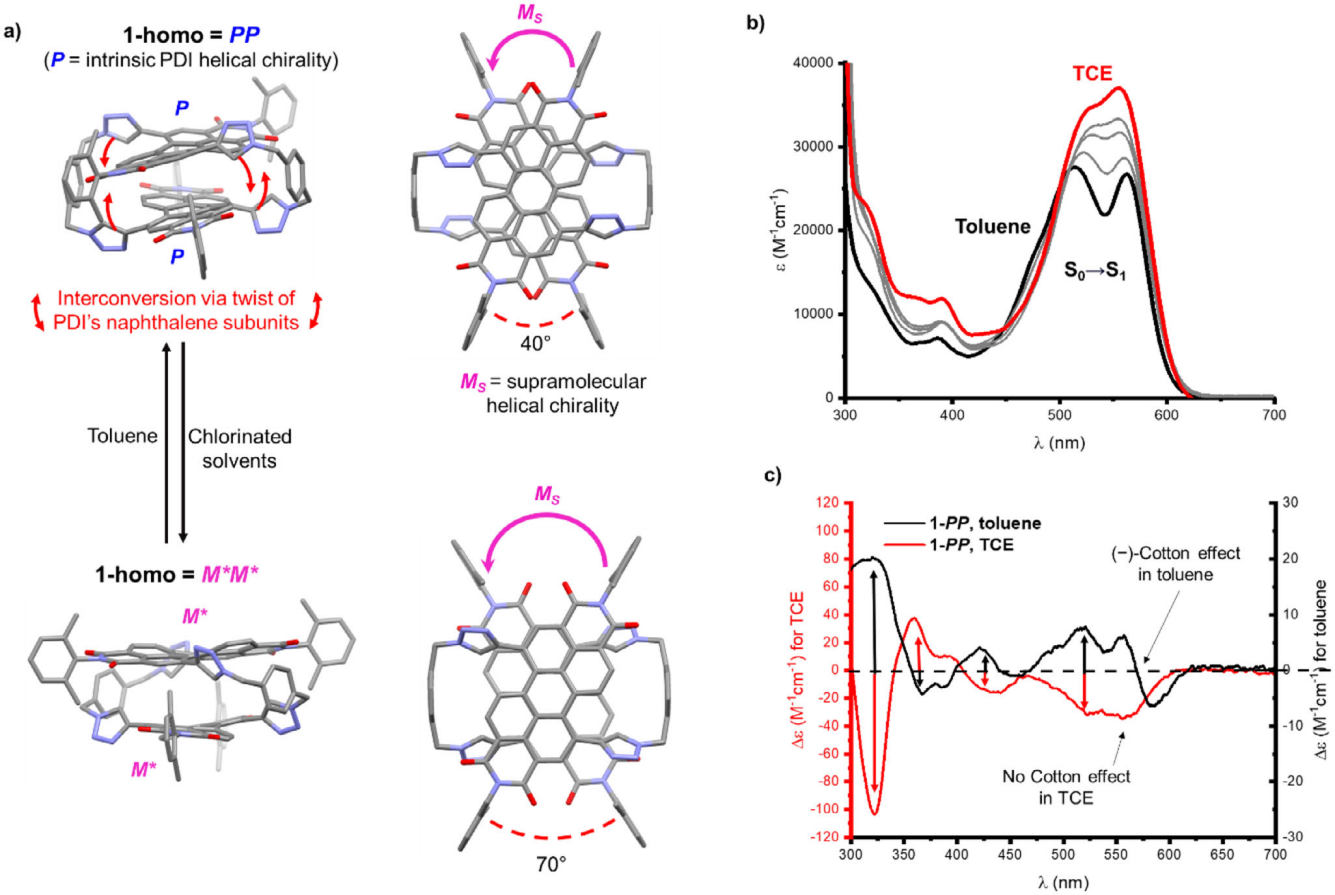
**a)** The likely conformation of **1‐homo** (**1‐*PP*** enantiomer shown) populated in toluene (top) and in chlorinated solvents (bottom), showing the intrinsic and supramolecular helicity of the bis‐PDI dimer. These are based on the two lowest energy conformations of **1‐homo** predicted by DFT (Supporting Information Section 8). **b)** UV–vis absorption spectra of **1‐homo** (10 µM) measured in neat toluene (black), TCE (red), and intermediate toluene:TCE solvent ratios (grey). **c)** CD spectra of **1‐homo** (**1‐*PP*** enantiomer,10 µM) in toluene (black) and TCE (red).

We first examined the conformation and intramolecular excitonic coupling of the homochiral PDI dimer **1‐homo** in nonpolar (toluene) and polar (chlorinated) solvents. These solvents were selected because they are known to impact the noncovalent interactions between PDI dyes.^[^ ^64^ ^]^ In toluene, UV‐vis and ^1^H NMR spectroscopy revealed that the conformation of **1‐homo** is that of a co‐facial π–π dimer (Figure 3a), which results in H‐type excitonic coupling between the PDI chromophores (Figure 3b).^[^ ^65^ ^]^ The supramolecular helicity of **1‐homo** is evident from the Cotton effect in the CD signal of the S_0_→S_1_ PDI absorption band (λ = 570 nm, Figure 2c), which arises from excitonic chirality in the macrocycle dimer. As expected, **1**‐***MM*** and **1**‐***PP*** exhibit opposite supramolecular helicity, and interestingly the intrinsic helical chirality of the twisted PDI (***M***/***P***) goes in hand with the opposite supramolecular helicity (***M***_s_/***P***_s_) of the co‐facial dimer, i.e., ***PP*** → ***M_s_*** and ***MM*** → ***P_s_***.^[^ ^59^ ^]^ This relationship appears to arise because it optimizes π–π contacts between parallel naphthalene sub‐units of the two PDIs and reduces steric clashing, as seen in a DFT optimized structure of macrocycle **1**‐***PP*** (Figure 3a).^[^ ^46^ ^]^

In chlorinated solvents, ^1^H NMR, UV‐vis (and CD – vide infra) spectroscopy reveal that **1‐homo** adopts a different conformation (Figure 3a‐c, S4‐1 and S4‐2). In line with our studies on a related bis‐PDI macrocycle,^[^ ^65^ ^]^ 1,1,2,2‐tetrachloroethane (TCE) weakens the π–π interactions in the dimer, causing an increase in the relative rotation of the two PDI units (Figure 3a) and hence a significant decrease in their intramolecular excitonic coupling, to generate two monomeric PDIs (Figure 3b,c, S4‐1 and S4‐2). The fluorescence quantum yield of **1‐homo** increases from Φ = 0.4 in toluene to Φ = 0.6 in TCE, reflecting the weaker H‐type coupling in the latter solvent.

**Figure 4:**
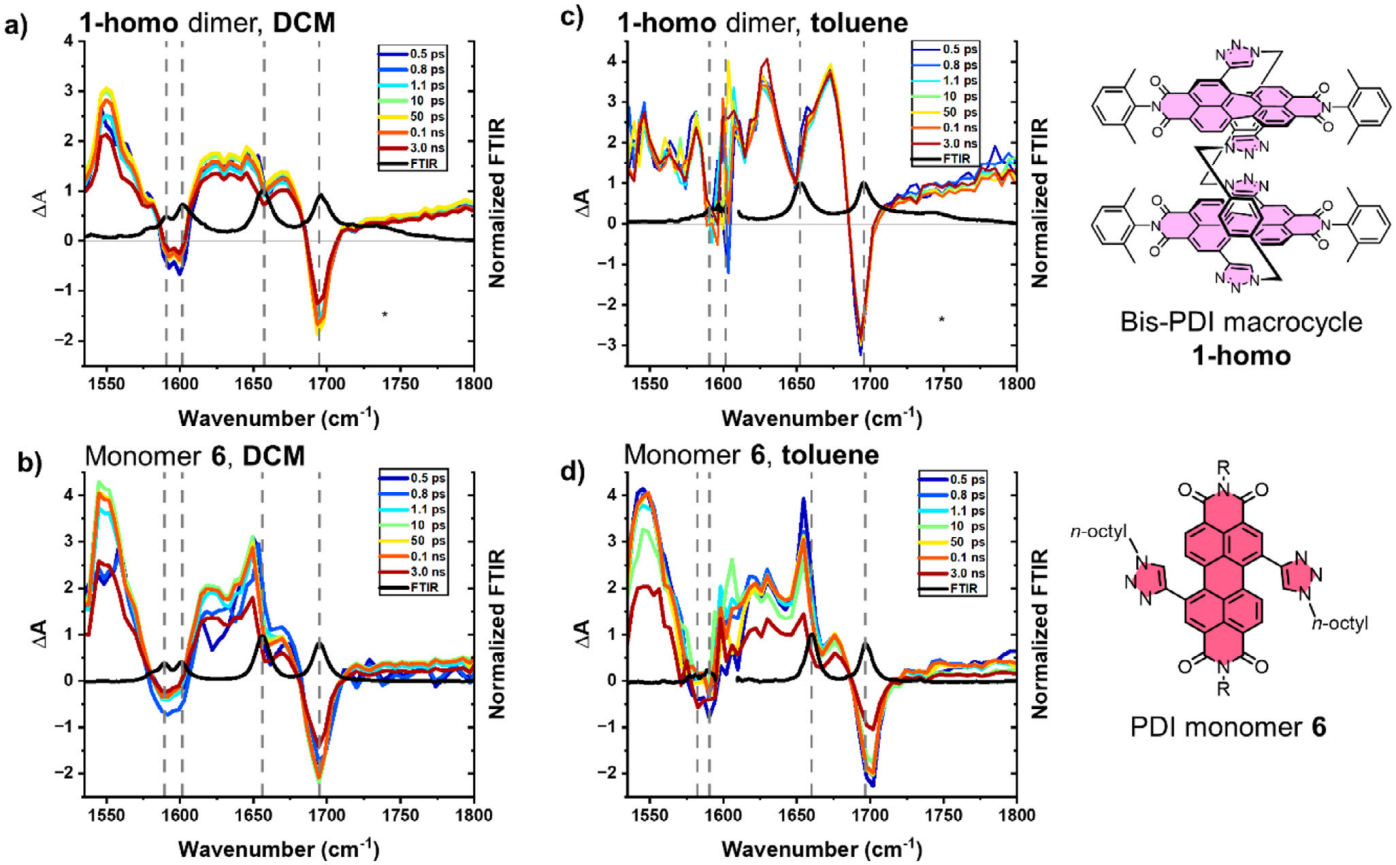
TRIR of the bis‐PDI macrocycle **1‐homo** (1 mM) and PDI monomer **6** (1 mM) recorded in DCM (**a** and **b**) and toluene (**c** and **d**), following excitation at 520 nm, 40 fs. FTIR spectra normalised to ν(CO) at 1652–1660 cm^−1^ are also shown (black line). Note that the region around 1600 cm^−1^ in toluene is obscured by IR absorbencies of the solvent.

The conformational change in **1‐homo** was further investigated by time‐resolved infrared (TRIR) spectroscopy (Figure 4). PDIs are excellent candidates for TRIR spectroscopy since their carbonyl groups are highly sensitive to changes in electron density distribution arising from changes in electronic coupling. For TRIR, dichloromethane (DCM) was used in place of TCE due to both solubility and IR‐absorption limitations.

Initially, the Fourier transform infrared (FTIR) spectra of **1‐homo** and a 1,7‐ditriazole PDI monomer **6** (Supporting Information Section 2) were measured in dry DCM and toluene, revealing four vibrational modes between 1500 cm^−1^ and 1800 cm^−1^ (Figure S7‐1 and Table S7‐1). The perylene ring breathing mode, ν(CC), appears at 1591 cm^−1^ in the macrocycle and 1589 cm^−1^ in the monomer, alongside two symmetric/antisymmetric combinations of carbonyl ν(CO) stretching vibrations between 1650 and 1696 cm^−1^ for both compounds. The ν(CO) of the monomer and the macrocycle are virtually identical in DCM. However, in toluene, a downward shift of the ν(CO)_asymm_ by 8 cm^−1^ (to 1652 cm^−1^), and of ν(CO)_sym_ by 2 cm^−1^ (to 1695 cm^−1^) is observed in the macrocycle **1‐homo** versus the monomer **6**, indicating a slight increase in the delocalisation in the former, which can be linked to the PDI–PDI excitonic coupling evidenced by UV‐vis and CD spectroscopy (Figure 3b,c). The ν(CO)_asymm_ in the macrocycle in toluene is also shifted by 4 cm^−1^ to lower frequency versus DCM, consistent with the solvent dependence of this coupling in the macrocycle dimer. For the final mode, a triazole stretching vibration, ν(CC), is observed in DCM at 1601/1602 cm^−1^ but is masked in toluene by solvent IR‐absorbencies.

A TRIR spectroscopic study was then conducted in the range from 1500 to 1800 cm^−1^ under 520 nm, 40 fs excitation, for both the PDI macrocyclic dimer **1‐homo** (Figure 4a,c) and monomer **6** (Figure 4b,d).^[^ ^66^, ^67^ ^]^ In all cases, TRIR spectra show instantaneous bleaches of the ground state vibrations, including ν(CO) stretches at ∼1650 cm^−1^ and 1700 cm^−1^, and the perylene core stretching vibration ν(CC) at around 1590 cm^−1^; all excited state features appear at lower frequency relative to their ground state parent bands. The excited state IR spectra (transients) for the monomer **6** in DCM and toluene are similar, albeit with a slightly different relative intensities of the transient peaks.

In contrast to PDI monomer **6**, the TRIR spectra for bis‐PDI macrocycle **1‐homo** in toluene are drastically different to that in DCM, providing supporting evidence for stronger intramolecular PDI–PDI interactions in toluene, in line with the previous ^1^H NMR, UV‐vis, and CD spectroscopic studies. Instead of a broad transient between 1600–1650 cm^−1^ observed in DCM, the TRIR spectra in toluene show two sharp transients shifted to lower frequency relative to the ground state bleaches: at 1630 cm^−1^ (bleach 1652.5 cm^−1^) and 1673 cm^−1^ (bleach 1695 cm^−1^). The shift is consistent with excess electron density being localised on the C = O groups, as documented for other aromatic acid imide anions.^[^ ^67^, ^68^ ^]^ The 1630 cm^−1^ transient is not pronounced in the TRIR spectrum of the macrocycle in DCM, potentially because the interactions between the two PDI units, which leads to a charge shift between them, is not efficient in DCM. Indeed, the higher intensity of the IR transients versus ground state bleaches in the macrocycle **1‐homo** in toluene (Figure 4c) versus DCM (Figure 4a) is consistent with a partial increase of electron density of the carbonyl groups, associated with an increased dipole moment. Moreover, the intense transient band at 1550 cm^−1^ associated with a ν(CC) in the excited state of the monomer in both solvents, and of the macrocycle in DCM, appears split for the macrocycle in toluene, again pointing toward coupling between the perylene cores. Finally, the broad IR transient seen in DCM is not present for the macrocycle in toluene, indicating that **1‐homo** populates a distinct and well‐defined conformation in the latter solvent.

The excited state dynamics of the monomer **6** in both solvents, and of the macrocycle **1‐homo** in DCM, are similar to one another, with a fast component reflecting potentially vibrational cooling of a few picoseconds, and a concomitant decrease in the ground state bleach and excited state absorbencies with a time constant of approximately 2 ns (Supporting Information Section 7). Conversely, the TRIR of **1‐homo** in toluene shows negligible ground state recovery on the timescale of the experiment but does demonstrate an apparent growth of the 1630 cm^−1^ feature attributed to an anion, on the timescale of approximately 6 ps, which could indicate an additional charge shift process in toluene.

### 1.3 Conformation‐dependent Photophysics of 1‐meso

**Figure 5:**
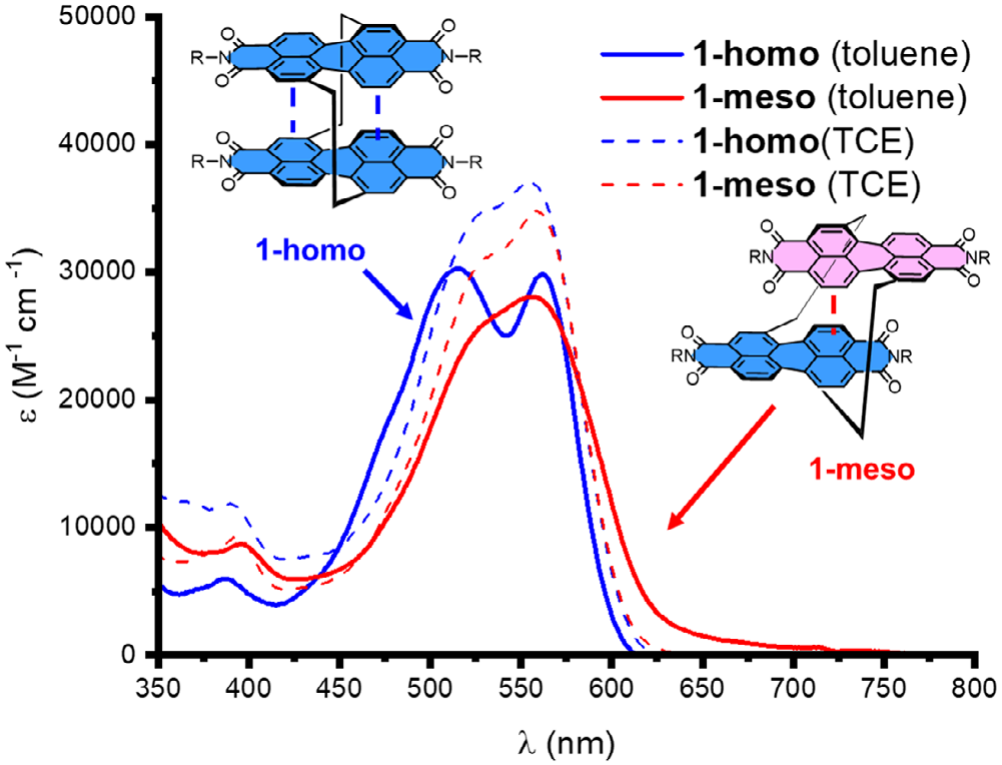
Comparison of the UV‐vis absorption spectra (10 µM) of **1‐meso** (red traces) and **1‐homo** (blue traces) in toluene (solid traces) and TCE (dotted traces) showing that coupling in the PDI dimer is dependent on chiral complementarity.

We next turned our attention to the meso isomer of the bis‐PDI macrocycle, to understand how the absence of chiral complementarity impacts the macrocycle's conformation and photophysical properties. In TCE, the ^1^H NMR (Figure 2b) and UV‐vis (Figure 5) spectra of **1‐meso** are comparable to **1‐homo**, indicative of a conformation in which the PDIs are only weakly interacting. Notably, the symmetrical ^1^H NMR spectrum of **1‐meso** is consistent with the triazole substituents eclipsing each other which generates the characteristic mirror plane of a meso isomer (Figure 5),^[^ ^61^ ^]^ in contrast to **1‐homo** where the triazole substituents are staggered (Figure 3a). Due to this mirror plane, there will be no preference for ***M_s_***/***P_s_*** supramolecular helicity in **1‐meso**.

The conformation of **1‐meso** changes upon the addition of toluene, with ^1^H NMR spectroscopy indicating a distinct conformation relative to **1‐homo** due to the shifting of PDI protons in the opposite direction (Figure S5‐1).^[^ ^69^ ^]^ From UV‐vis spectroscopy (Figure 5), 1 **‐meso** appears to exhibit weaker (H‐type) excitonic coupling in toluene than **1‐homo**, reflecting the mismatched PDI π‐surfaces of opposite chirality which will hinder co‐facial π–π stacking. Indeed, a new red‐shifted shoulder on the S_0_→S_1_ PDI absorption band of **1‐meso** in toluene suggests a long‐axis^[^ ^42^ ^]^ and/or rotational displacement^[^ ^70^, ^71^ ^]^ of the PDI units (Figure 5). Relative to **1‐homo**, the fluorescence emission of **1‐meso** is red‐shifted and has a lower quantum yield in toluene (Φ = 0.2 versus 0.4), which is also consistent with a distinct excited state for the ***MP*** dimer.

### 1.4 CD Switching

Our next goal was to explore the impact of changes in chiral conformation and excitonic coupling on the CD spectra of **1‐homo**. A solvent‐into‐solvent titration of 1,1,2,2‐tetrachloroethane (TCE) into toluene was monitored by CD spectroscopy and revealed two major changes in the spectrum of ***1‐MM*** (Figures 3c and S4‐1) Firstly, TCE causes a loss of the S_0_→S_1_ Cotton effect in ***1‐MM***, revealing that the excitonic chirality, the sign of which is connected to the supramolecular helicity of the PDI dimer, is only present in toluene. Secondly, there is a general reversal in the sign of the peaks across the rest of the CD spectrum (λ < 550 nm), which covers the higher energy PDI transitions (S_0_→S_n>1_). This chiroptical switching is intriguing considering that ***1‐MM*** cannot undergo enantiomerization to ***1‐PP*** (via an intramolecular somersault) because it is configurationally stable.

The first change (loss of S_0_→S_1_ Cotton effect, Figure 3c) is explained by the increased rotational displacement of the PDIs of **1‐*homo*** in TCE (Figure 3a), which leads to significantly weaker intramolecular excitonic coupling (Figure 3b).^[^ ^65^ ^]^ In other words, while the supramolecular helicity remains the same in both solvents (i.e., ***1‐MM*** maintains ***P_s_***), it only has an impact on the CD spectrum in toluene because there is excitonic coupling between the PDI chromophores. There is a linear correlation between the CD signal (Δε at λ = 526 nm) and excitonic coupling (ε_0–0_/ε_0–1_) in ***1‐MM***, which highlights the connection between excitonic chirality and chiroptical activity (Figure S4‐1).^[^ ^72^ ^]^

**Figure 6:**
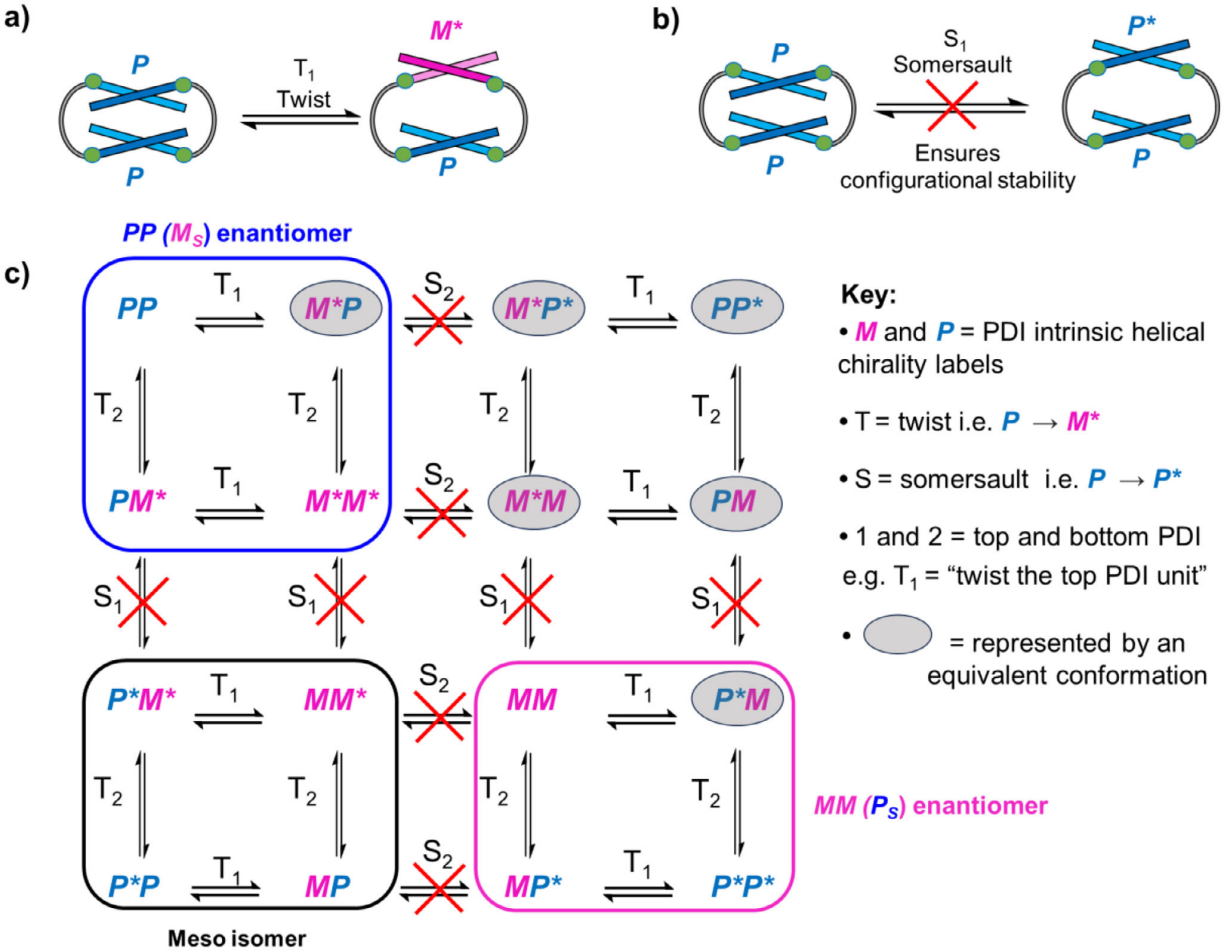
Cartoon depiction of **a)** a “twist” and **b)** a “somersault” of one PDI core in a bay‐connected bis‐PDI macrocycle (**1**). **c)** Scheme outlining the 16 possible chiral conformations of **1‐homo** when considering the intrinsic helical chirality and the relative positioning of the triazole substituents in both PDI units. The (*) indicates that the bay substituents point into the macrocycle's cavity, reflecting the pseudo‐enantiomeric relationship between, e.g., ***P*** and M*.

The second change (general inversion of the CD spectrum at λ < 550 nm, Figure 3c) is also connected to the conformational change of **1‐*homo***. The larger rotational displacement of the PDIs in chlorinated solvent is accommodated by an inversion of the twist of the two naphthalene subunits of each chromophore (Figure 3a), a conformational change which inverts the intrinsic helicity of both PDIs and so explains the general inversion of the CD spectrum. This inversion of PDI intrinsic helicity is most notable from the change in orientation of the triazole heterocycles in the PDI's (1,7) bay positions (Figure 6a, ‘Twist’). While in toluene these bay substituents are positioned outside the macrocycle's cavity to facilitate intramolecular π–π interactions, in chlorinated solvents the triazole heterocycles are directed into the cavity. We label this (pseudo‐enantiomeric) conformational change as ***MM*** → P*P* (or ***PP*** → M*M*), where the (*) indicates that the bay substituents point into the macrocycle's cavity (Figures 3a and 5a). The assignment of PDI intrinsic chirality in chlorinated solvents was also confirmed by comparison with the CD spectra of the enantiomers of a previous 1,7‐ditriazole PDI monomer.^[^ ^73^ ^]^ The ***MM*** and P*P* conformers are not mirror images of each other and so neither are their CD spectra (Figure 3c), meaning that chiroptical switching is achieved without interconversion between two enantiomers. From DFT calculations, the two lowest energy conformers of **1‐homo** are found to be ***MM***/***PP*** and P*P*/M*M* (Supporting Information Section 8), with the former predicted to be lower in energy in toluene, consistent with experiments.

Overall, the intrinsic PDI helicity (***M***/***P***) and bay‐substituent orientation (*) leads to 16 possible chiral conformations for a bay‐connected bis‐PDI macrocycle (Figure 6c), which are all related by the intramolecular twisting or somersaulting of the two PDI units (Figure 6a,b).^[^ ^74^ ^]^ For macrocycle **1**, where both PDI units are equivalent, six of these conformers are identical (e.g., ***PM*** = ***MP***). Furthermore, the symmetric ^1^H NMR spectra of **1‐homo** and **1‐meso** show that nonsymmetrical conformers such as P*P and ***M*** * ***P*** are not isolated/populated, most likely because these would require eclipsing (P*P) or staggered (***M*** * ***P***) bay substituents which are sterically unfavored. Importantly, since the bis‐PDI macrocycle **1‐homo** is configurational stable, the somersaulting mechanism is inhibited and so the switching between equal and opposite CD spectra cannot occur, i.e., ***PP*** → ***MM*** enantiomerisation (Figure 6c). Instead, **1‐homo** switches between distinct chiral conformers without the risk of racemization and due to an inversion of the PDI's core‐twist (e.g., ***MM*** → P*P* pseudo‐enantiomerisation), thereby realising a robust chiroptical switch that is operated using an achiral solvent stimulus (Figures 3c and 6c).

### 1.5 Multiphoton Circularly Polarized Luminescence Switching

**Figure 7:**
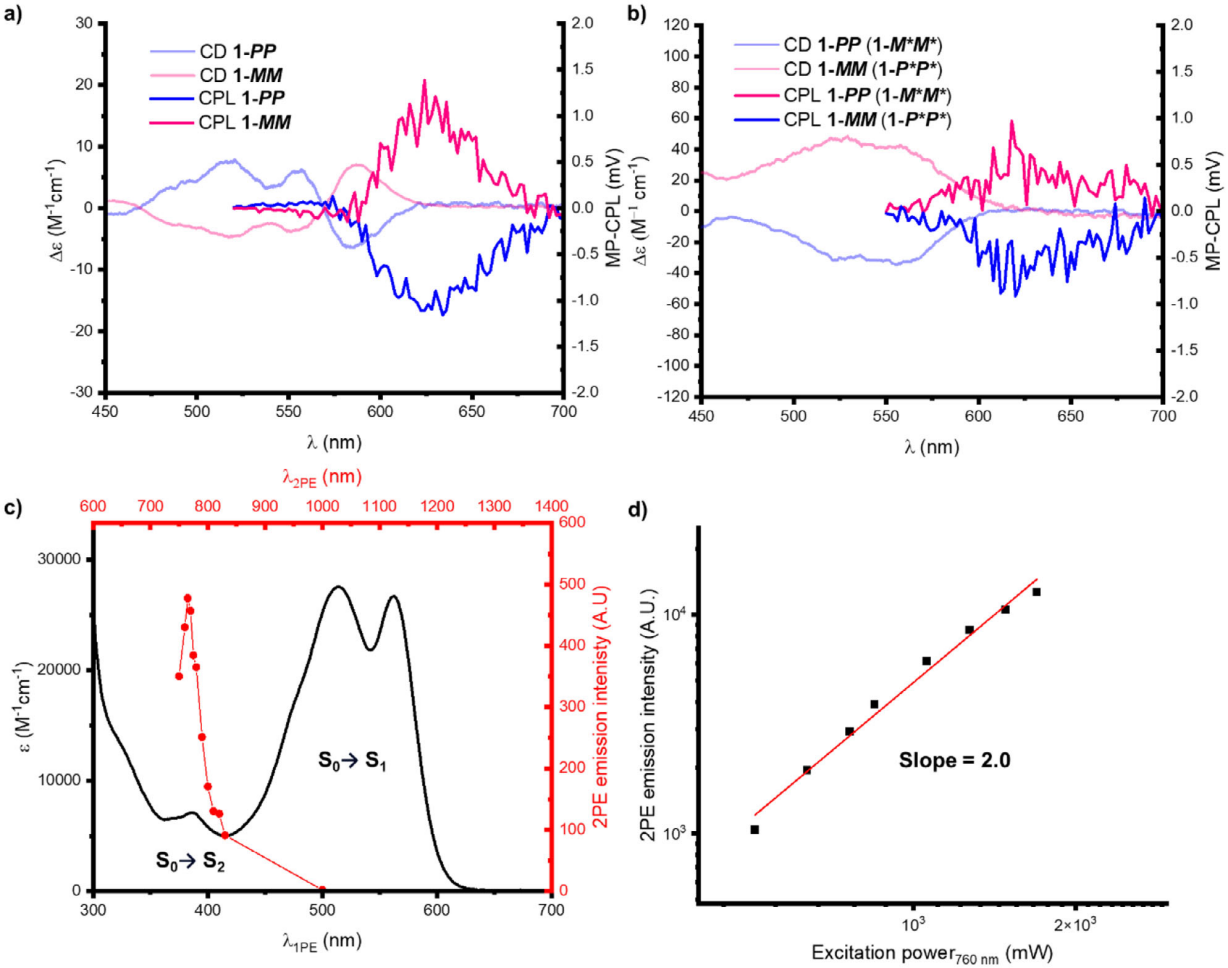
a) CD and MP‐CPL (λ_ex_ = 760 nm, nonsmoothed) spectra for **1‐*MM*** and **1‐*PP*** in toluene (10 µM). b) CD and MP‐CPL (λ_ex_ = 760 nm, nonsmoothed) spectra for **1‐*MM*** and **1‐*PP*** in TCE (10 µM). **c**) UV‐vis absorption (black trace) and two‐photon excitation spectrum (red dots, λ_em_ = 610 nm) for macrocycle **1‐homo** in toluene (10 µM). d) Excitation power dependence of the two‐photon excitation (2PE) luminescence emission intensity (λ_ex_ = 760 nm), slope = 2.0 ± 0.1, *σ*_2_ = 388.

The enantiomers of **1‐homo** are configurationally stable, emissive, and have a relatively high two‐photon absorption cross section (σ_2_ = 388 Göppert‐Mayer [GM]**1‐homo** at 760 nm, where 1 GM = 10^−50^ cm^4^s/photon, Figure 7d, Supporting Information Section 6) making them excellent candidates for multiphoton excitation circularly polarized luminescence (MP‐CPL). The MP excitation of **1‐homo** was shown to be a two‐photon event at λ = 760 nm as the laser power dependence of the two‐photon induced emission at this excitation wavelength has a slope of ∼ 2 on a logarithmic scale (Figure 7d).^[^ ^37^, ^38^ ^]^ Therefore, we performed MP‐CPL on the **1‐homo** enantiomers ***1‐MM*** and ***1‐PP*** using two‐photon excitation.

Consistent with their enantiomeric relationship, ***1‐MM*** and ***1‐PP*** gave mirror image MP‐CPL spectra in toluene (Figure 7a). Importantly, the emission bands are identical to those measured by single‐photon excitation CPL with the same g_lum_ values (Table 1, Figures S6‐2,3,4), indicating that emission occurs from the same excited state. The multiphoton absorption spectrum suggests excitation occurs only via the S_0_→S_2_ transition (Figure 7c), which is consistent with multiphoton studies into centrosymmetric molecules such as PDIs,^[^ ^75^ ^]^ where the S_0_→S_1_ transition is usually one‐photon allowed and two‐photon forbidden. Additionally, the relatively high value of σ_2_ compared to other PDIs could be explained by there being a strong one‐photon absorption band (i.e., the S_0_→S_1,_ > 450 nm) close to the two‐photon absorption laser wavelength (760 nm), which can act as an “intermediate state” that facilitates the two‐photon absorption process.^[^ ^76^ ^]^

**Table 1:** Dissymmetry factors (g_abs_ and g_lum_) for **1**‐***PP***. Errors in g_abs_ and g_lum_ are ± 2 × 10^−4^ and ± 5 × 10^−5^, respectively.

	g_abs_ (× 10⁻^3^)			
Solvent	λ < 450 nm	λ > 450 nm	g_lum_ (× 10⁻^3^)	g_lum_ (MP‐CPL) (× 10⁻^3^)
TCE	−3 [365 nm]	−1 [558 nm]	−1	−1
Toluene	+1 [358 nm]	−1 [585 nm]	−3	−3

We also measured both MP‐CPL and CPL spectra for the enantiomers of **1‐homo** in TCE and revealed that the change in the macrocycle's conformation switches the amplitude of (MP‐)CPL (Figure 7b), with the g_lum_ being three times larger in toluene than TCE (Table 1). Interestingly, the sign of the (MP‐)CPL signal is not inverted upon changing the solvent, with **1**‐P*P* maintaining a positive signal in TCE (originally **1**‐***MM*** in toluene) while **1**‐M*M* (originally **1**‐***PP*** in toluene) maintains a negative signal (Figure 7a,b). Therefore, the sign of the (MP‐)CPL spectrum matches the sign of the lowest energy branch of the CD spectrum in each solvent (Table 1), meaning that the contributions of intrinsic and supramolecular helicity which defined the ground state chirality also explain the excited state chirality.

Considering TCE first, negligible intramolecular excitonic coupling means that the two PDIs in **1‐homo** are essentially monomeric in this solvent (Figure 3). As such, the sign of the MP‐CPL signal is dictated by the intrinsic helicity of the PDI units. Indeed, **1**‐P*P* (or **1**‐M*M*) gives a positive (or negative) CPL signal, in line with the ***P*** (***M***) intrinsic helicity of other monomeric core‐twisted PDI enantiomers.^[^ ^77^ ^]^ In toluene however, the excitonic coupling between PDIs is stronger (Figure 3), meaning that the supramolecular helicity of the PDI dimer now defines the sign of the CPL signal.^[^ ^78^ ^]^ The ***P_s_*** (or ***M_s_***) supramolecular helicity of the **1**‐***MM*** (**1**‐***PP***) PDI dimer leads to a positive (negative) Cotton effect in the CD spectrum, which corresponds to the positive (negative) sign of the CPL signal.

Further evidence for the changing importance of intrinsic and supramolecular helicity in **1‐homo** comes from comparing the g_abs_ and g_lum_ values of **1**‐***PP*** (**1**‐M*M*) in different solvents (Table 1).^[^ ^79^, ^80^, ^81^, ^82^ ^]^ In TCE, g_lum_ = g_abs_, which is consistent with monomeric PDI units, whereby intrinsic PDI helicity defines the ground and excited state chirality, which are similar due to the PDI's relatively rigid π‐scaffold.^[^ ^77^ ^]^ However, for **1**‐***PP*** in toluene, g_lum_ > g_abs_, which highlights the increasingly dynamic nature of chirality arising from (noncovalent) supramolecular helicity relative to the (covalent) intrinsic helicity of a core‐twisted PDI chromophore. Importantly, this also demonstrates the potential of excitonic chirality in a supramolecular helical PDI dimer to increase the g_lum_ beyond that of monomeric core‐twisted PDIs (i.e., **1‐homo** in toluene versus TCE), an effect which forms the basis of the MP‐CPL amplitude switching of **1‐homo** seen here (Figure 7a,b).

### 1.6 Summary and Conclusions

We have developed a novel chiral bis‐PDI macrocycle (**1**) which combines configurational stability with conformational flexibility. The former property enabled the isolation of both homochiral enantiomers (**1‐homo**) and the meso isomer (**1**‐**meso**), establishing a connection between chiral complementarity and excitonic coupling. While **1‐homo** exhibits H‐type coupling between the two PDI units, the mismatched chirality of the PDI π‐surfaces of **1**‐**meso** results in weaker coupling. Furthermore, **1‐homo** can be switched between different homochiral conformations, as characterised by their distinct photophysics, including by time‐resolved infrared spectroscopy which provides additional support for solvent‐dependent intramolecular interactions between the PDI units. This conformational flexibility of **1‐homo** tunes intramolecular excitonic coupling in the PDI dimer, enabling us to unravel and understand the important roles of PDI intrinsic and supramolecular helicity in defining the chiroptical properties of **1‐homo**, both in ground and excited states.

Despite the configurational stability of **1‐homo**, there is a general inversion of the macrocycle's CD spectrum on switching the solvent from toluene to TCE. This phenomenon is explained by a concomitant inversion of the intrinsic helicity of both PDI units (e.g., ***1‐MM*** → 1‐P*P* pseudo‐enantiomerisation) and a loss of excitonic chirality due to weaker coupling in the PDI dimer. The latter means that both PDIs can be considered monomeric and so there is no contribution to the CD spectrum from their supramolecular helicity. This underpins the design of +/− chiroptical switches^[^ ^16^, ^17^, ^18^, ^19^, ^20^, ^21^, ^22^, ^47^ ^]^ in the mold of **1‐homo**, whereby an achiral stimulus triggers an inversion in the chiral conformation (e.g., ***P*** → M*), without the requirement for enantiomerization (i.e., ***P*** → ***M***) using a chiral stimulus.

The analysis of **1‐homo** in the excited state was performed by MP‐CPL spectroscopy, which is of interest for future CPL‐based imaging and sensing applications. Notably, the sign of the MP‐CPL signal does not change with a change in solvent. This shows that the chirality of the ground state dictates the chirality of the excited state, with supramolecular or intrinsic helicity governing the sign of the MP‐CPL when the PDIs are coupled or uncoupled, respectively. Instead, MP‐CPL switching by **1‐homo** is delivered through g_lum_ amplification in toluene compared to TCE. This highlights the important role of excitonic chirality to boosting (MP‐)CPL and how, compared to intrinsic chirality, supramolecular helicity may lead to greater differences between ground and excited state chiral conformations. Therefore, excitonic coupling between organic dyes is an important marker for efficacious chiroptical materials and may be used for chiroptical switching, including of both the sign and signal strength.

## 2 Supporting Information

Synthetic procedures, characterisation data, additional spectra, DFT optimized structures, and experimental and computational methods can be found in the Supporting Information. Additional references cited within the Supporting Information.^[^ ^83^, ^84^, ^85^, ^86^, ^87^, ^88^, ^89^, ^90^, ^91^, ^92^, ^93^, ^94^, ^95^ ^]^

## Conflict of Interest

The authors declare no conflict of interest.

## Supporting information



Supporting Information

Supporting Information

## Data Availability

The data that support the findings of this study are available in the supplementary material of this article.
