# Supplementary material for: Circular Dichroism and Multiphoton Circularly Polarized Luminescence Switching Using a Bis‐perylene Diimide Macrocycle
Source: Chemistry. 2025 Aug 22;31(53):e01734. doi: 10.1002/chem.202501734 (PMC12451419; doi:10.1002/chem.202501734)
Supplement: Supplementary file 1 — Supporting Information [file CHEM-31-e01734-s001.pdf]

Circular Dichroism and Multiphoton Circularly Polarised Luminescence Switching Using a Bis-perylene Diimide Macrocycle

S. E. Penty,[a,b] M. V. Appleby,[c] M. Zwijnenburg,[d] D. J. Black,[b] D. Hartmann,[a] D. Chekulaev,[c] J. Weinstein,*[,c] R. Pal*[,b] & T. A. Barendt*[,a]

Addresses:

[a]School of Chemistry, University of Birmingham, Edgbaston, Birmingham, B15 2TT, United Kingdom.

[b]Department of Chemistry, University of Durham, South Road, Durham, DH1 3LE, United Kingdom.

[c]School of Mathematical and Physical Sciences, University of Sheffield, Dainton Building, 13 Brook Hill, Sheffield S3 7HF.

[d]Department of Chemistry, University College London, 20 Gordon Street, London WC1H 0AJ, United Kingdom.

Supporting Information

Contents

1. Materials and methods 2
2. Synthesis and characterisation 6
3. Stereoisomer analysis 16
4. Conformational switching studies for 1-homo 20
5. Conformational switching studies for 1-meso 23
6. (Multiphoton) CPL spectroscopy 25
7. Time-resolved infrared (TRIR) spectroscopy 28
8. Density Functional Theory Calculations 30
9. References 31

1. Materials and methods

General

All commercial solvents and reagents were used as purchased, unless otherwise stated. Anhydrous solvents were degassed with N_2 and dried using an Innovative Technology PureSolv MD 5 solvent purification system. $Cu(MeCN)_4 \cdot PF_6$ was stored in a desiccator. Tris((1-benzyl-4-triazolyl)methyl)amine (TBTA) was prepared following a literature procedure.[1] Water was distilled and microfiltered using an ELGA DV 35 Purelab water purification system. Chromatography was undertaken using silica gel (particle size: 40-63 μm) or preparative TLC plates (20 × 20 cm, 1 cm silica thickness). Dry Toluene and DCM were prepared via the Grubbs service at the University of Sheffield.

Analytical techniques

^1H and ^{13}C NMR spectra were recorded using Bruker AVIII400 (400 MHz), Bruker AV NEO 400 (400 MHz) Bruker AV NEO 500 (500 MHz, with cryoprobe). Mass spectra were recorded using a Bruker UltrafleXtreme MALDI-TOF mass spectrometer or a Waters Synapt G2-S mass spectrometer for high resolution MS-ESI. Semi-preparatory HPLC was carried out using a COSMOSIL Buckyprep 250 x 10 mm column. Chiral HPLC was carried out using a Phenomenex i-Amylose-1, 250 x 4.6 mm (analytical) or a Phenomenex i-Amylose-1, 250 x 10 mm (semi-preparatory).

UV-vis absorption spectroscopy

All steady state electronic absorption spectra were recorded at a concentration of 10 μM (unless otherwise stated) at 298 K. For UV-vis-NIR spectroscopy a Shimadzu UV-3600i Plus spectrophotometer was used, with a wavelength accuracy ± 0.2 nm in the UV-vis range and absorbance accuracy ± 0.002 Abs. Quartz cuvettes with 1 cm path length were used.

Fluorescence spectroscopy

All fluorescence spectra were recorded at a concentration of 10 μM at 298 K. For fluorescence spectroscopy a Jasco FP8500 was used with emission and excitation wavelength accuracies ± 1.0 nm. The detector base sensitivity is 8500:1. Quartz cuvettes with 1 cm path length were used.

Quantum yield measurements

Absolute fluorescence quantum yields were obtained on an Edinburgh Instruments FLS1000 photoluminescence spectrometer fitted with an integrating sphere. All samples were recorded in toluene at a 1 μM with a 7 - 8 nm excitation slit and 0.1 - 0.2 nm emission slit width. Experiments were carried out in solution using 1 cm path length quartz cuvettes with four transparent polished faces.

Circular dichroism (CD) spectroscopy

Circular dichroism (CD) spectra were recorded on a Jasco J-1500 CD spectrophotometer with a wavelength accuracy ± 0.2 nm (250 to 500 nm), ± 0.5 nm (500 to 800 nm) and a CD root mean square noise < 0.007 mdeg (500 nm). A quartz cuvette with 1 mm path length was used. The spectra were recorded at a concentration of 10 μM.

Circularly polarised luminescence (CPL) spectroscopy

CPL was measured with a home-built (modular) spectrometer.[2] The excitation source was a broad band (200 – 1000 nm) laser- driven light source EQ 99 (Elliot Scientific). The excitation wavelength was selected by feeding the broadband light into an Acton SP-2155 monochromator (Princeton Instruments, 7 μm slit); the collimated light was focused into the sample cell (1 cm quartz cuvette,

temperature controlled at 20 ºC). Sample PL emission was collected perpendicular to the excitation direction with a lens (f = 150 mm). The emission was fed through a photoelastic modulator (PEM) (Hinds Series II/FS42AA) and through a linear sheet polariser (Comar). The light was then focused into a second scanning monochromator (Acton SP-2155, 7 µm slit) and subsequently on to a photomultiplier tube (PMT) (Hamamatsu H10723 series). The detection of the CPL signal was achieved using the field modulation lock-in technique. The electronic signal from the PMT was fed into a lock-in amplifier (Hinds Instruments Signaloc Model 2100). The reference signal for the lock-in detection was provided by the PEM control unit. The monchromators, PEM control unit and lock-in amplifier were interfaced to a desktop PC and controlled by a custom-written Labview graphic user interface. The lock-in amplifier provided two signals, an AC signal corresponding to (I_L- I_R) and a DC signal corresponding to (I_L + I_R) after background subtraction. The emission dissymmetry factor was therefore readily obtained from the experimental data, as 2 AC/DC.

Spectral calibration of the scanning monochromator was performed using a Hg-Ar calibration lamp (Ocean Optics, HAL-200). A correction factor for the wavelength dependence of the detection system was constructed using a calibrated lamp (Ocean Optics). The measured raw data was subsequently corrected using this correction factor. The validation of the CPL detection systems was achieved using light emitting diodes (LEDs) at various emission wavelengths. The LED was mounted in the sample holder and the light from the LED was fed through a broad band polarising filter and λ/4 plate (Ocean Optics) to generate circularly polarised light. Prior to all measurements, the λ/4 plate and a LED were used to set the phase of the lock-in amplifier correctly. The emission spectra were recorded with 2 nm step size and the slits of the detection monochromator were set to a slit width (vide supra) corresponding to a spectral resolution of 3.5 nm. CPL spectra (as well as total emission spectra) were obtained through an averaging procedure of several scans.

Multiphoton (MP) CPL spectroscopy

Two photon CPL spectroscopy was achieved by coupling (beam routing using mirrors, Thor Labs BB1E03) a tunable femtosecond pulsed laser (680 – 1300 nm, Coherent Discovery TPC, 100 fs, 80 MHz) to the pre-existing CPL spectrometer described above.

Multiphoton (MP) spectroscopy

Two photon spectroscopy was achieved as described in previous publications[3] by perpendicularly mounting an Ocean Optics HR2000Pro (2048-pixel linear CCD Sony ILX5 chip, 200 µm slit, H3 grating, 350 – 850 nm spectral region) spectrometer as a 'third arm' to the Discovery TPC laser. The laser beam was focused onto the centre of the 1 cm path sample holder (Thor labs CVH100) by a dedicated ultrafast laser lens (Edmund Optics 11711, 50 mm focal length). The spectrometer has also been equipped with a perpendicularly mounted 365 nm LED (nichia, 1W) and been operated using a modified version of the above-mentioned custom time resolved detection and accumulation algorithm written in Labview2013 program. In order to eliminate unwanted artefacts associated with stray light from MP excitation each spectrometer have been equipped with a rotating filter wheel (Thor Labs, CFW6) housing an LP420 (Comar Optics, for 365 nm UVLED excitation) and SP650 and SP700 (Edmund Optics, 8472 and 8474 for MP excitation) filters.

Fourier transform infrared (FTIR) spectroscopy

FTIR spectra were recorded with a Thermo Nicolet instrument with a resolution of 2 cm^{-1}. Samples were recorded in a Harrick Cell with CaF_2 windows (2 mm thickness) with a typical path length of 500 µm.

Time-resolved infrared (TRIR) spectroscopy

TRIR was carried out in the Lord porter Laboratory at the University of Sheffield. A Ti:Sapphire regenerative amplifier (Spitfire ACE PA-40, Spectra-Physics) provided 800 nm pulses (40 fs FWHM, 10 KHz, 1.2 mJ). The amplifier was seeded by 800 nm pulses (25 fs FWHM, 84 MHz) generated by a Ti:Sapphire oscillator (Mai Tai, SpectraPhysics). Both amplification stages of the Spitfire ACE were pumped by two Nd:YLF lasers (Empower, Spectra-Physics). The Ti:Sapphire regenerative amplifier (Spitfire ACE PA-40, Spectra-Physics) was used to pump a commercial optical parametric amplifier (TOPAS, Light Conversion), which provided tunable mid-IR pulses (2300-10000 cm^{-1}, typical FWHM of ~280 cm^{-1}) by difference frequency generation in AgS. After the optical parametric amplifier (OPA), the probe beam passed through a Ge long pass filter and was collimated to a beam diameter of 10 cm in a two-mirror telescope. A wire-grid polariser (Thorlabs) was used in conjunction with a tuneable $\lambda/2$ wave plate (AlphasLas) to attenuate the power of the probe pulses. Before the sample, the probe was split into a probe and refence beam using a 50:50 Ge beam splitter. Both beams passed through the sample, the reference was positioned such that it does not overlap with the UV-vis pump.

The 520 nm (FWHM 10 nm) pump pulses were generated with a second commercial OPA (TOPAS, Light Conversion) also pumped by the same amplifier. A mechanical delay-stage (Thorlabs) was used to set the delay time between the pump and probe pulses, by extending the pump path in relation to the probe. This gave an experimental window of 3 ns. The pump repetition rate was set to 5 kHz using an optical chopper (Thorlabs), data was collected at 10 kHz this allows for the collection of pump-probe spectra. The power of the pump pulses was attenuated before the sample using a variable attenuation neutral density filter wheel. The relative polarisation of the pump was set to magic angle (54.7°) relative to the probe polarisation using a tuneable $\lambda/2$ wave plate (Thorlabs) to reduce anisotropic effects in the measurements.

The IR probe (~ 1 mW) and reference (~1 mW) were focussed onto the sample position using a gold off-axis parabolic mirror (f = 211 mm) to a spot size of ~150x150 μm. The UV-vis pump was focussed and moved to overlap onto the probe position at the sample using an aluminium 90 degree off-axis parabolic mirror (f = 100 mm) to a spot-size of ~300x300 μm. The sample was mounted on a mechanical z stage to adjust the sample position in the focal point of the beam. After the probe and refence passed through the sample, the beams are collected on another parabolic mirror. The reference has an independent mirror path to control both beams into the detector. The beams are split by an additional 50:50 Ge beam splitter.

The split beams were focussed into two identical spectrometers (f = 320 mm, Horiba iHR320). A grating of 50 g/mm was used, giving a spectral resolution of 6, 3 and 2 cm^{-1} respectively. The probe and reference spectra were recorded employing two liquid nitrogen cooled HgCdTe (MCT) array detectors (Infrared Systems). Each detector had 128 pixels split into 2 lines; 96 pixels for the probe and 32 for the reference.

Home-built software (LabVIEW) was used for data collection and processing the raw spectra to pump-probe TRIR spectra. A multichannel referencing scheme was used to improve the signal-to-noise ratio. Measurements were calculated from the average of 5 scans with 2 s averaged per time point (ca. 10000 on/off pulse pairs).

Samples were prepared in a Harrick Scientific IR cell with 2mm CaF_2 windows. The path length was adjusted using a PFTE spacer (500 μm). Samples were raster-scanned at the sample position using a motorised x-y stage to minimise photodegradation. Calibration of the probe axis was achieved by fitting the spectral resolution (nm $pixel^{-1}$) and central wavelength in a least-squares minimisation

routine, where an absorption spectrum measured in-situ was fit to the FT-IR spectrum of neat dioxane in a 100 μm cell.

2. Synthesis and characterisation

The synthesis of bis-PDI macrocycle **1** was carried out as shown in **Scheme S1**. Compound **4** was prepared according to a literature procedure[4] as a mixture of mono-, bis-, tris- and tetra-bromo isomers that could not be separated using chromatography at this stage due to poor solubility.

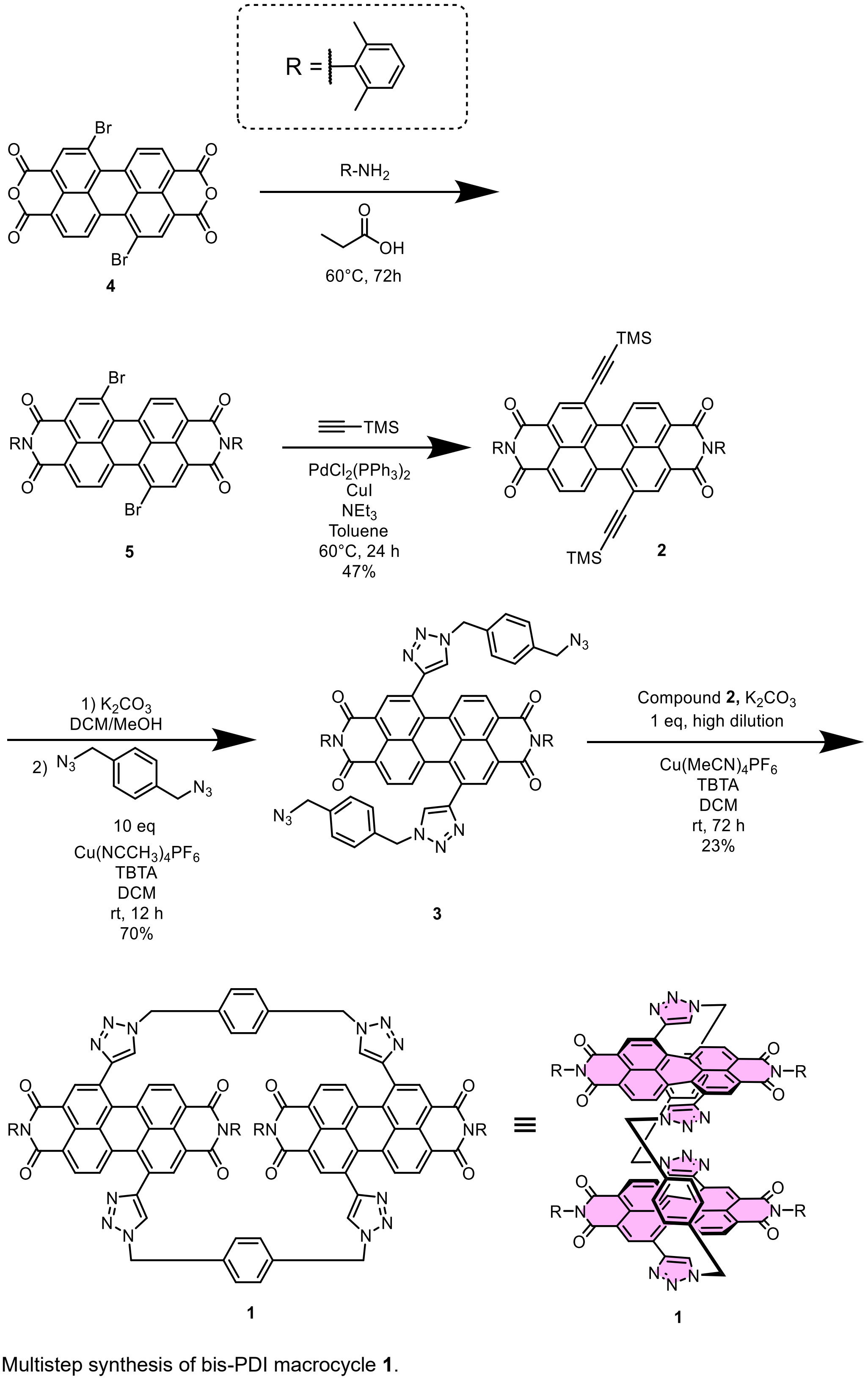

Scheme S1: Multistep synthesis of bis-PDI macrocycle **1**.

Bis-bromo PDI 5[4]

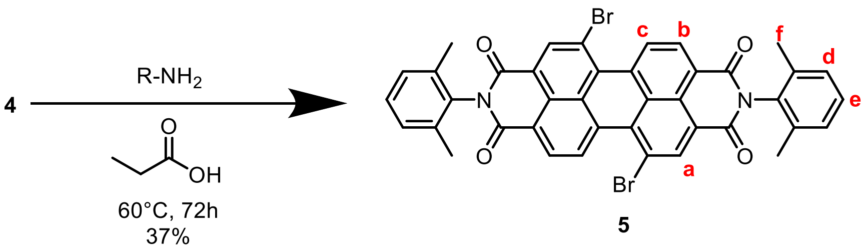

Compound **4** (a mixture of mono-, bis-, tris- and tetra-bromo perylenetetracarboxylic dianhydride, 5 g) was added to a flask. To this was added propionic acid (100 mL) and 2,6-dimethylaniline (6 mL, 50 mmol, 5.5 equiv). The mixture was purged with nitrogen and heated at 60 °C for 72 h. The reaction mixture was then cooled to rt, which yielded a red precipitate. This was washed with water (200 mL) and methanol (50 mL). The resulting red residue was then purified by silica gel flash column chromatography (1:1 n-hexane:CH_2Cl_2), affording compound **5**[5] as a mixture of 1,6 and 1,7 bis-bromo isomers (2.54 g, 3.4 mmol, 37% yield if compound **4** was pure bis-bromo perylenetetracarboxylic dianhydride).

^{1}H NMR (500 MHz, Chloroform-*d*, 1,7-isomer) δ 9.58 (dd, J = 8.1, 3.2 Hz, $2H_a$), 9.02 (s, $2H_b$), 8.81 (dd, J = 8.1, 3.6 Hz, $2H_c$), 7.33-7.25 (m, $6H_{d-e}$), 2.18 (s, $12H_f$), agreeing with literature reports. [5]

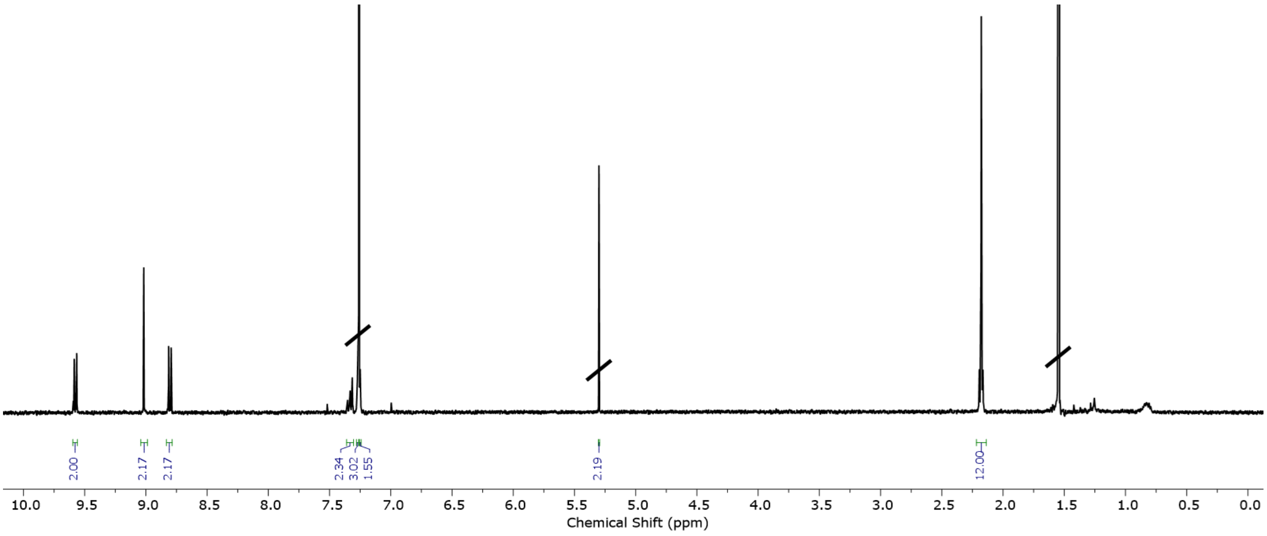

^{1}H NMR spectrum of compound **5** (chloroform-d, 298 K, 500 MHz)

TMS-protected bis-alkyne PDI 2

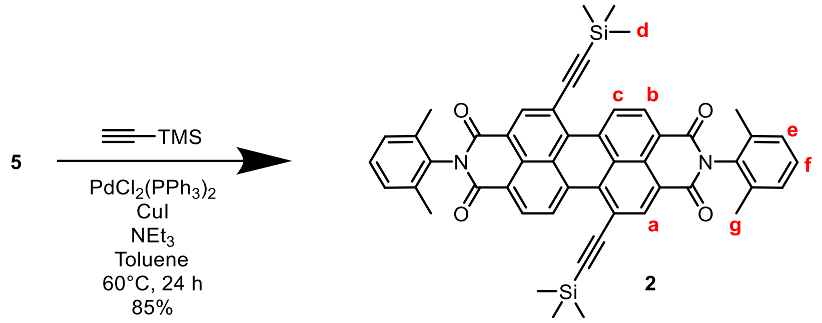

To a solution of bis-bromo PDI **5** (580 mg, 644 μmol) in 1:1 dry NEt_3-toluene (250mL) under a nitrogen atmosphere was added $Pd(PPh_3)_2Cl_2$ (27 mg, 39 μmol, 0.06 equiv), CuI (14 mg, 73 μmol, 0.11 equiv) and trimethylsilylacetylene (316 mg, 458 μL, 3.22 mmol, 5 equiv). The mixture was thoroughly de-gassed with nitrogen and stirred at 60 °C for 24 h. The solvent mixture was then removed in vacuo. The resulting residue was then re-dissolved in DCM (100 mL) and washed with 1 M HCl (50 mL) and water (3 x 50 mL); dried over anhydrous $MgSO_4$ and concentrated to dryness in vacuo. The resulting residue was purified by silica gel flash column chromatography (1:1 n-hexane:DCM) affording the title compound as a red solid as a mixture of 1,6 and 1,7 bis-bromo regioisomers (515 mg, 550 μmol, 85%).

^{1}H NMR (500 MHz, Chloroform-*d,* 1,7 isomer) δ 10.26 (d, J = 8.2 Hz, 2H_b), 8.91 (d, J = 7.8 Hz, 2H_a), 8.74 (d, J = 8.2 Hz, 2H_c), 7.33– 7.24 (m, 6H_{e-f}), 2.18 (s, 12H_g), 0.39 (s, 18H_d).

^{13}C NMR (126 MHz, Chloroform-*d*) δ 162.70, 162.38, 138.96, 138.62, 135.66, 135.00, 134.67, 133.59, 131.11, 129.25, 128.79, 128.74, 128.54, 128.10, 128.02, 127.80, 123.44, 122.32, 120.38, 106.56, 105.53, 18.10, 18.05.

HRMS (ESI) (m/z) calculated for $C_{50}H_{43}N_2O_4Si_2$ $[M+H]^+$ 791.2761, found 791.2773.

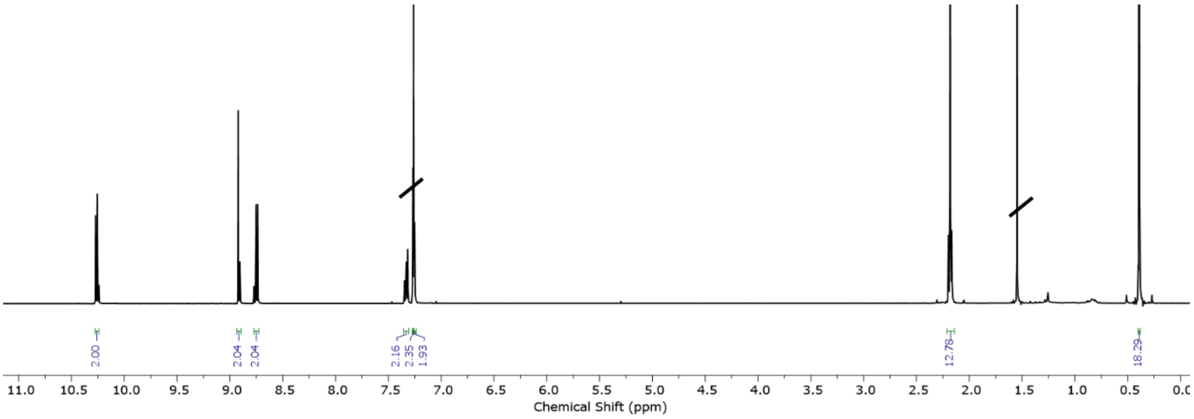

^{1}H NMR spectrum of compound **2** (chloroform-d, 298 K, 500 MHz).

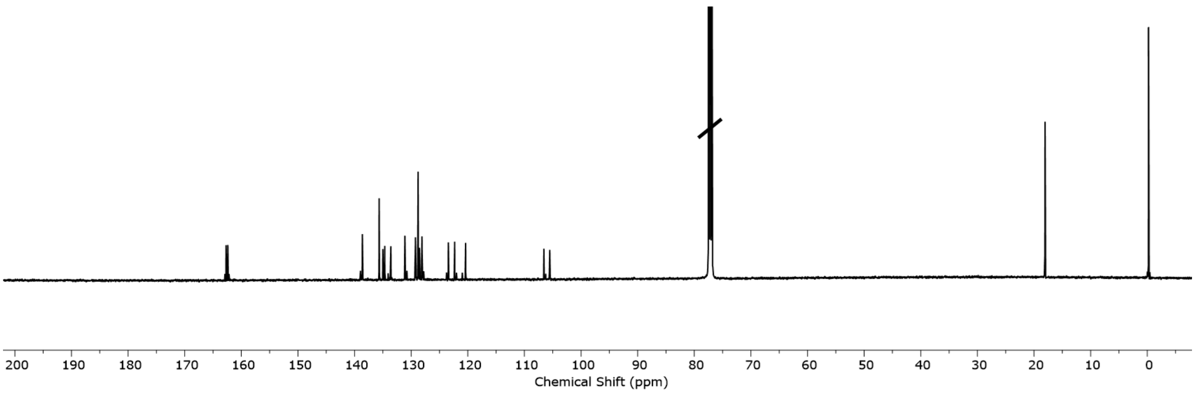

^{13}C NMR spectrum of compound **2** (chloroform-d, 298 K, 126 MHz)

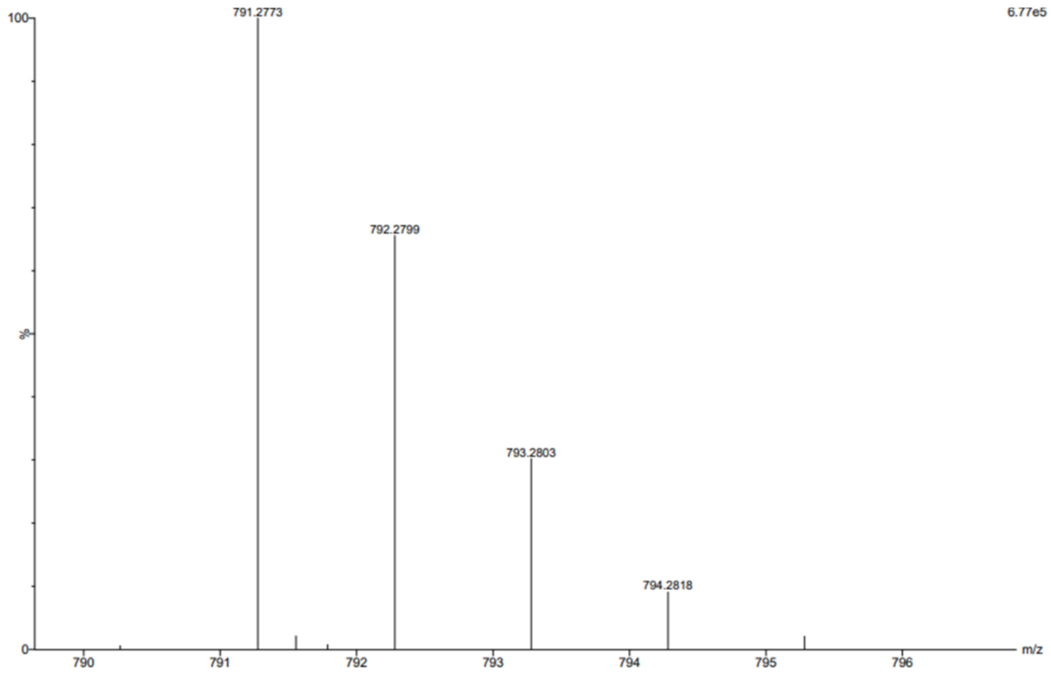

Observed ESI MS data for compound **2**.

Acyclic bis-triazole PDI 3

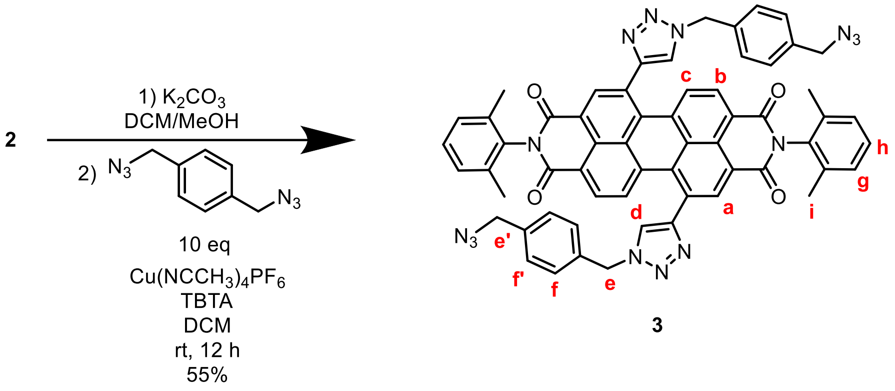

To a solution of TMS-protected bis-alkyne PDI **2** (155 mg, 194 μmol) in DCM (20 ml) was added K_2CO_3 (30 mg) in MeOH (10 ml). The mixture was stirred at rt for 3 min, and completion of the reaction was confirmed by TLC. The solution was then washed with water (2 x 30 mL) and brine (30 ml). The organic layer was then dried over anhydrous $MgSO_4$ and concentrated to dryness in vacuo to afford the deprotected PDI bis-alkyne which was used immediately without further purification. This PDI bis-alkyne was immediately re-dissolved in dry DCM (30 mL). To this was added 1,4-bis(azidomethyl)benzene (prepared via a literature procedure,[6] 182 mg, 971 μmol, 5 equiv) and tris((1-benzyl-4- triazolyl)methyl)amine (TBTA)[1] (20 mg, 39 μmol, 0.2 equiv). The solution was then de-gassed with argon. The copper (I) catalyst $Cu(CH_3CN)_4PF_6$ (15 mg, 39 μmol, 0.2 equiv) was then added and the solution was once again de-gassed with argon. The reaction was stirred at rt for 12 h. The solvent was then removed in vacuo. The resulting residue was purified by silica gel flash column chromatography (1:199 MeOH-DCM) affording the title compound as a purple solid as a mixture of 1,6 and 1,7 bis-bromo regioisomers (110 mg, 108 μmol, 55%).

^{1}H NMR (500 MHz, Chloroform-*d,* 1,7 isomer) δ 8.74 (s, $2H_a$), 8.24 (d, *J* = 8.0 Hz, $2H_b$), 7.93 (d, *J* = 8.0 Hz, $2H_c$), 7.72 (s, $2H_d$), 7.43 – 7.21 (m, $10H_{f,g,h}$), 5.67 (d, *J* = 2.2 Hz, $4H_e$), 4.37 (d, *J* = 2.4 Hz, $4H_{e'}$), 2.14 (s, $12H_i$).

^{13}C NMR (126 MHz, Chloroform-*d*) δ 162.49, 162.44, 148.20, 136.77, 135.47, 135.32, 135.01, 134.42, 133.95, 133.50, 130.19, 129.41, 129.30, 129.22, 129.11, 129.08, 128.91, 128.65, 128.52, 128.48, 122.68, 122.49, 122.00, 54.27, 54.17, 17.89

HRMS (MALDI-MS) (m/z) calculated for $C_{60}H_{41}N_{14}O_4$ [M-H] 1021.3435, found 1021.4322.

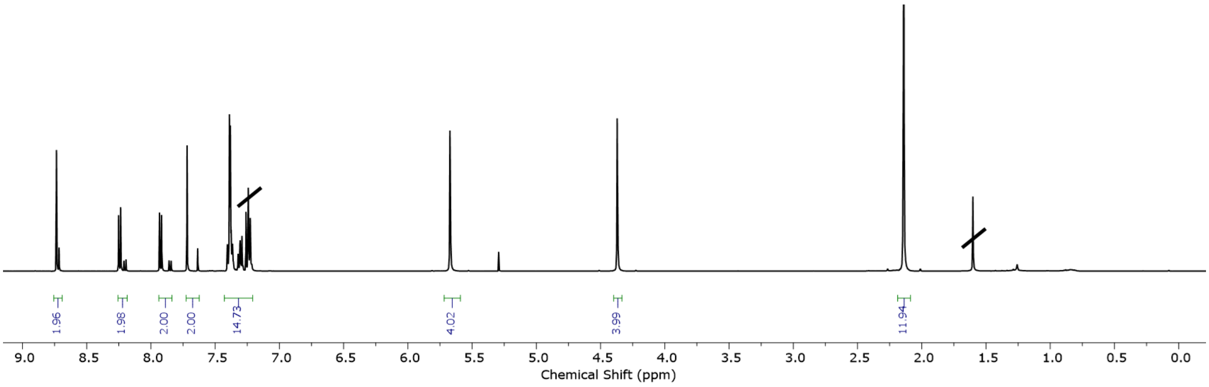

^{1}H NMR spectrum of compound **3** (chloroform-*d*, 298 K, 500 MHz).

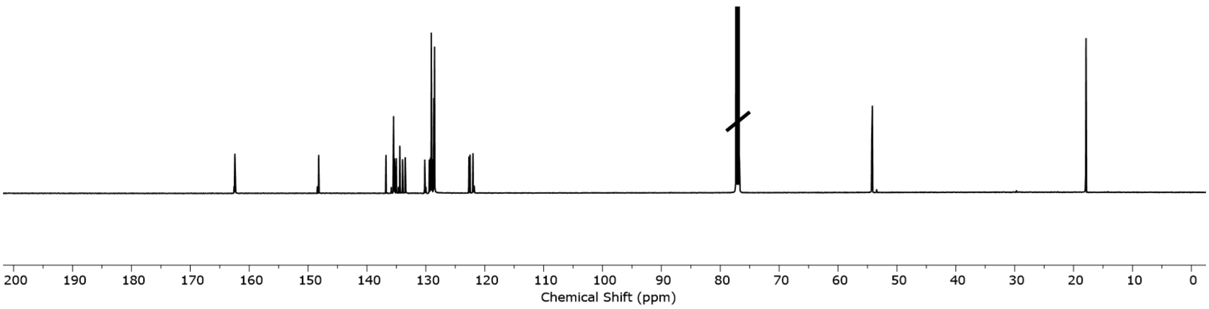

^{13}C NMR spectrum of compound **3** (chloroform-*d*, 298 K, 126 MHz).

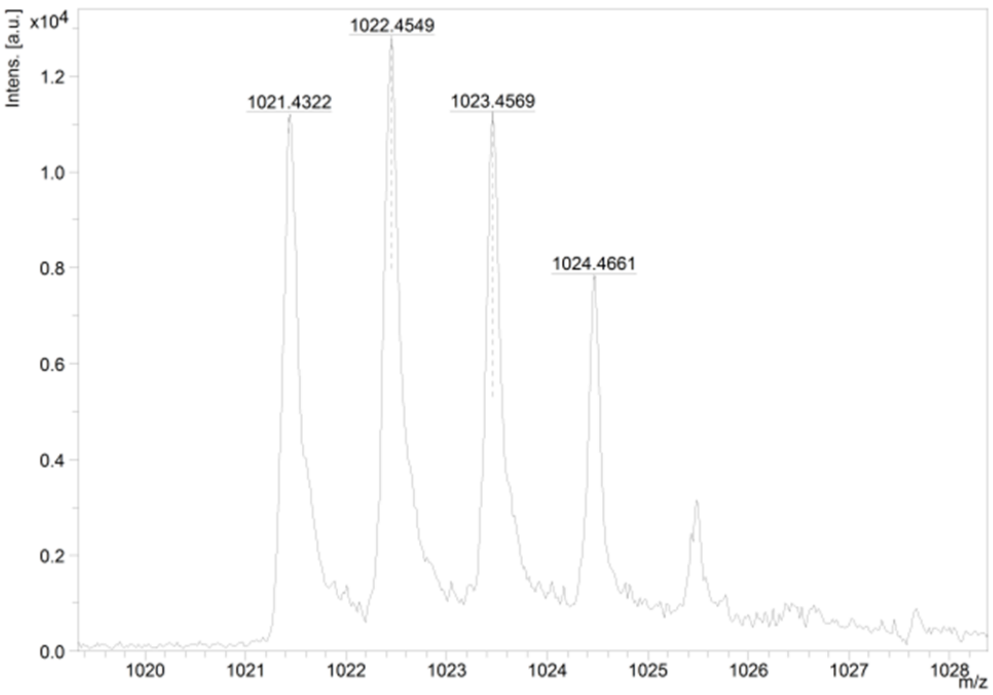

Observed (bottom) MALDI-MS data for compound **3**.

Bis-PDI macrocycle 1-homo/1-meso

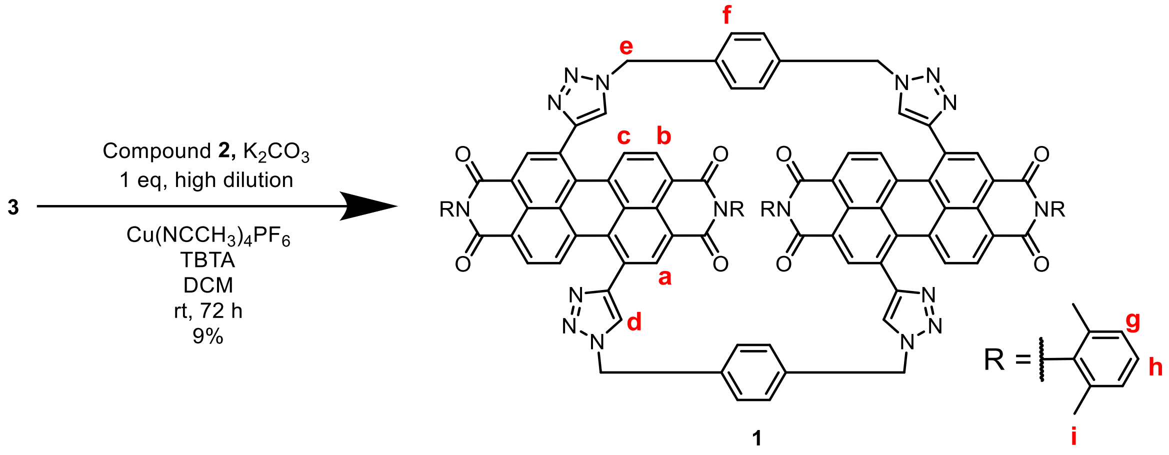

TMS-protected bis-alkyne PDI **2** (77 mg, 97 µmol) was dissolved in DCM (20 mL). To this was added K_2CO_3 (20 mg) in MeOH (10 mL). The reaction was monitored by TLC. Upon completion the reaction mixture was thoroughly washed with water in a separating funnel (3 x 100 mL) and dried with $MgSO_4$ to yield crude deprotected bis-alkyne PDI in DCM, which was used immediately without further purification due to its tendency to aggregate and crash out of solution over time. This was added to a flask, along with acyclic bis-triazole PDI **3** (99 mg, 97 µmol, 1 equiv), tris((1-benzyl-4-triazolyl)methyl)amine (TBTA) (21 mg, 39 µmol, 0.4 eq) and a further 350 mL of DCM. The reaction mixture was thoroughly de-gassed with N_2. The copper catalyst $Cu(CH_3CN)_4PF_6$ (14 mg, 39 µmol, 0.4 equiv) was then added and the reaction mixture was thoroughly de-gassed again. The reaction was stirred at rt for 36 h and monitored by TLC (2:98 MeOH-DCM). The solvent was then removed *in vacuo*. The resulting residue was dissolved in DCM and filtered through cotton wool to remove insoluble side products (proposed to be extended oligomeric species) and the filtrate was then purified by HPLC (COSMOSIL Buckyprep 250 x 10 mm, eluted with a gradient going from 1:1 (v/v) DCM:n-hexane eluent to 7:3:1 (v/v/v) DCM:n-hexane:isopropanol) affording the title compounds (pure 1,7-regioisomer) as purple solids (giving a combined total of 15 mg [97 µmol, 9%] of **1-homo** and **1-meso**, isolated in a 3:2 ratio).

^{1}H NMR (homochiral stereoisomer, 400 MHz, 343K, TCE-d_2) δ 8.76 (s, 4H_a), 8.33 (d, J = 8.1 Hz, 4H_b), 7.95 (d, J = 8.2 Hz, 4H_c), 7.41 – 7.21 (m, 24$H_{d,f,g,h}$), 5.73 (d, J = 15.2 Hz, 4H_e), 5.62 (d, J = 15.2 Hz, 4H_e), 2.17 (d, J = 6.6 Hz, 12H_i), 2.04 (s, 12H_i).

^{13}C NMR (101 MHz, TCE-d_2) δ 162.54, 162.17, 148.11, 136.07, 135.99, 135.57, 135.23, 134.72, 133.73, 133.69, 130.23, 129.76, 129.24, 129.07, 129.02, 128.74, 122.99, 122.93, 121.41, 120.60, 74.45, 74.17, 73.90, 54.30, 18.13, 17.94.

HRMS (MALDI-MS) (m/z) calculated for $C_{104}H_{68}N_{16}O_8$ [M-H] 1667.5327, found 1667.5328.

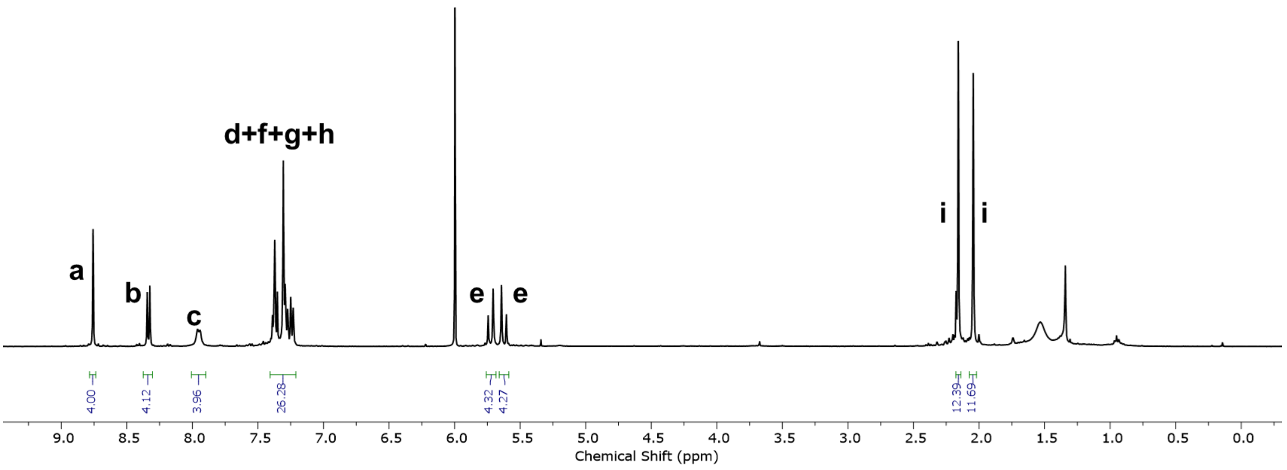

^1H NMR spectrum of macrocycle **1** (homochiral diastereomer, TCE-d_2, 343 K, 400 MHz).

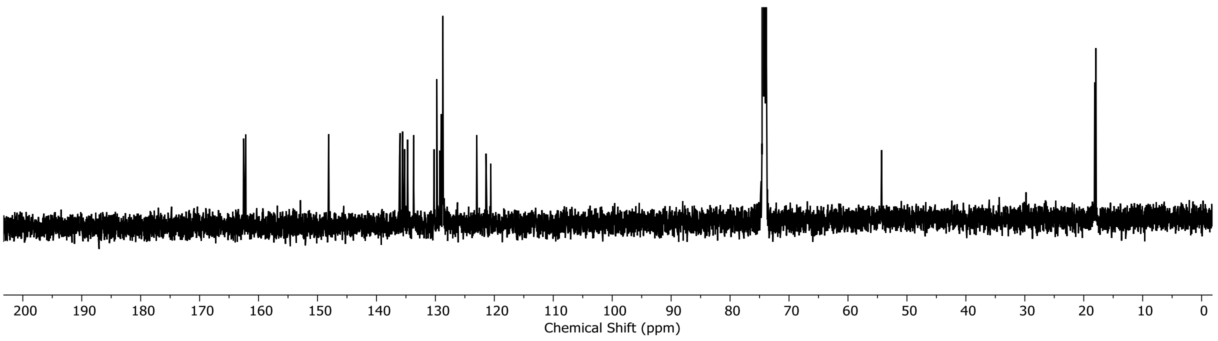

^{13}C NMR spectrum of macrocycle **1** (homochiral diastereomer, TCE-d_2, 343 K, 400 MHz)

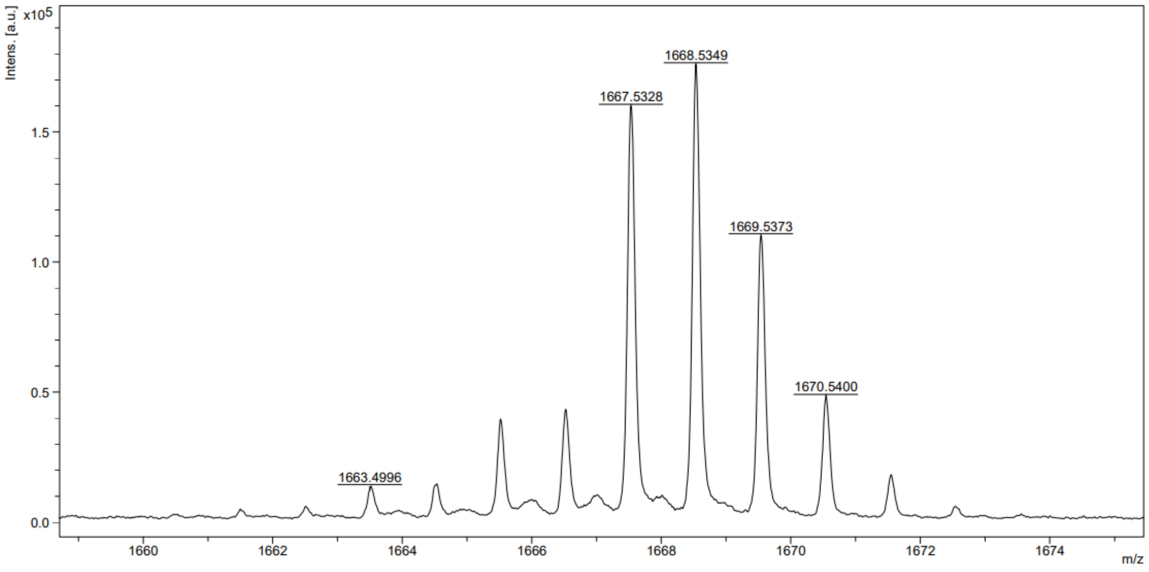

Observed MALDI-MS data for macrocycle **1** (homochiral diastereomer).

1,7-Ditriazole PDI monomer 6

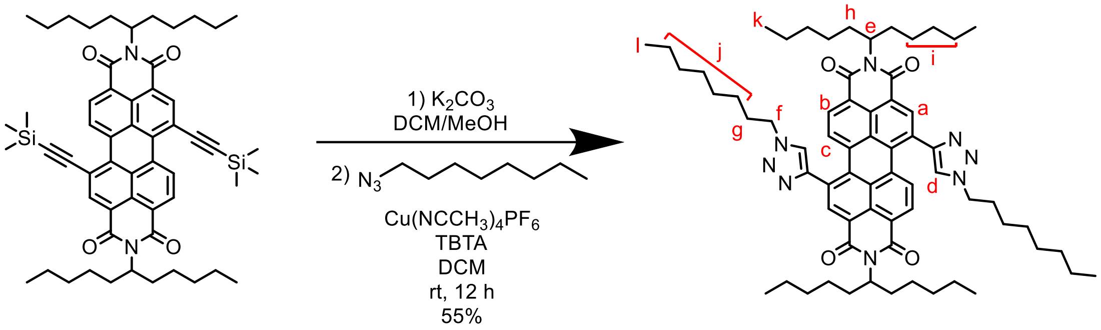

6

To a round-bottom flask equipped with a stirrer bar was added di(trimethylsilylacetylene)-C11-PDI (prepared according to literature procedures,[7] 212 mg, 0.238 mmol) and dissolved in 30 mL of a 2:1 mixture of CH_2Cl_2:MeOH. To this was added K_2CO_3 (118 mg, 0.857 mmol, 3.6 equiv) and the mixture stirred for 15 minutes, after which TLC (2:1 $PE_{40\text{-}60}$:CH_2Cl_2) indicated full deprotection. The solution was diluted with 20 mL of CH_2Cl_2, and washed with H_2O (2x 25 mL) and brine (1x 50 mL), dried ($MgSO_4$), filtered and reduced *in vacuo*. The crude, deprotected PDI was used straight in the next step without further purification. To the deprotected PDI was then added n-octyl azide (184 mg, 5 equiv) and TBTA (25 mg, 20 mol%), followed by 20 mL of CH_2Cl_2. The reaction mixture was degassed by sparging with N_2. Then, $Cu(MeCN)_4PF_6$ (18 mg, 20 mol%) was added and the mixture left stirring for 48 hours. The solvent was subsequently removed and the residue purified by flash column chromatography (SiO_2, 2% acetone/CH_2Cl_2) to yield a purple solid as the 1,7 regioisomerically pure title compound (169 mg, 0.160 mmol, 57%).

^{1}H NMR (400 MHz, $CDCl_3$) δ/ppm 8.67 (d, J = 16.0 Hz, 2H, **b**), 8.22 (d, J = 13.3 Hz, 2H, **a**), 7.93 (d, J = 8.1 Hz, 2H, **c**), 7.76 (s, 2H, **d**), 5.15 (s, 2H, **e**), 4.49 (t, J = 7.2 Hz, 4H, **f**), 2.22 (dtd, J = 14.3, 9.8, 4.0 Hz, 4H, **h'**), 2.00 (p, J = 7.6 Hz, 4H, **g**), 1.90 – 1.73 (m, 4H, **h''**), 1.46 – 1.14 (m, 44H, **i** & **j**), 0.93 – 0.78 (m, 18H, **k** & **l**).

^{13}C NMR (101 MHz, $CDCl_3$) δ/ppm 164.60, 163.48, 147.98, 135.58, 134.74, 133.56, 130.37, 129.61, 129.20, 128.53, 123.22, 122.50, 121.76, 54.78, 50.95, 32.41, 31.85, 30.49, 29.17, 29.04, 26.66, 26.62, 22.71, 22.67, 14.19, 14.16.

HRMS (ESI$^+$) found 1057.7051, $[M+H]^+$ requires 1057.7002.

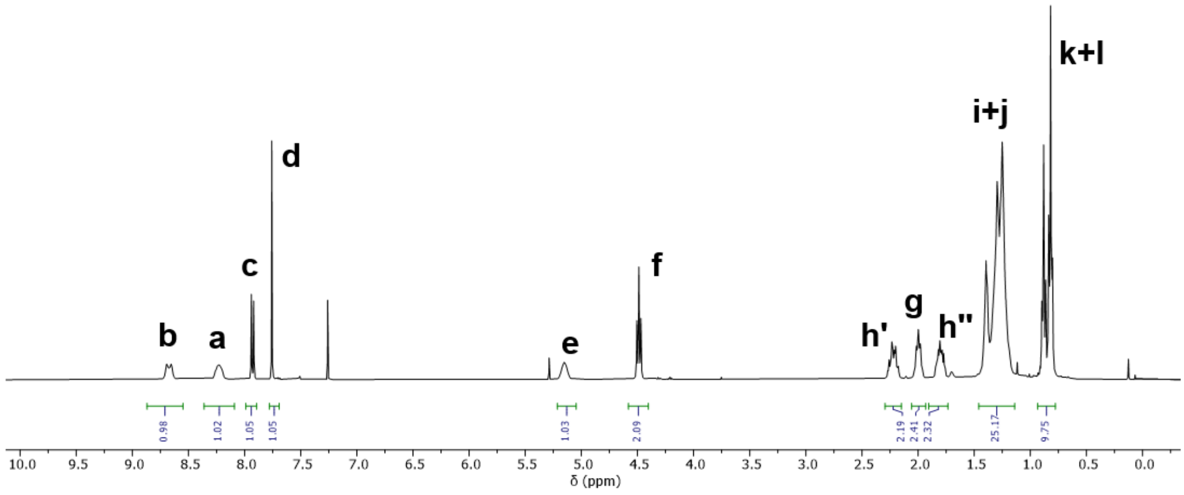

^{1}H NMR spectrum of acyclic monomer **6** ($CDCl_3$, 298 K, 400 MHz).

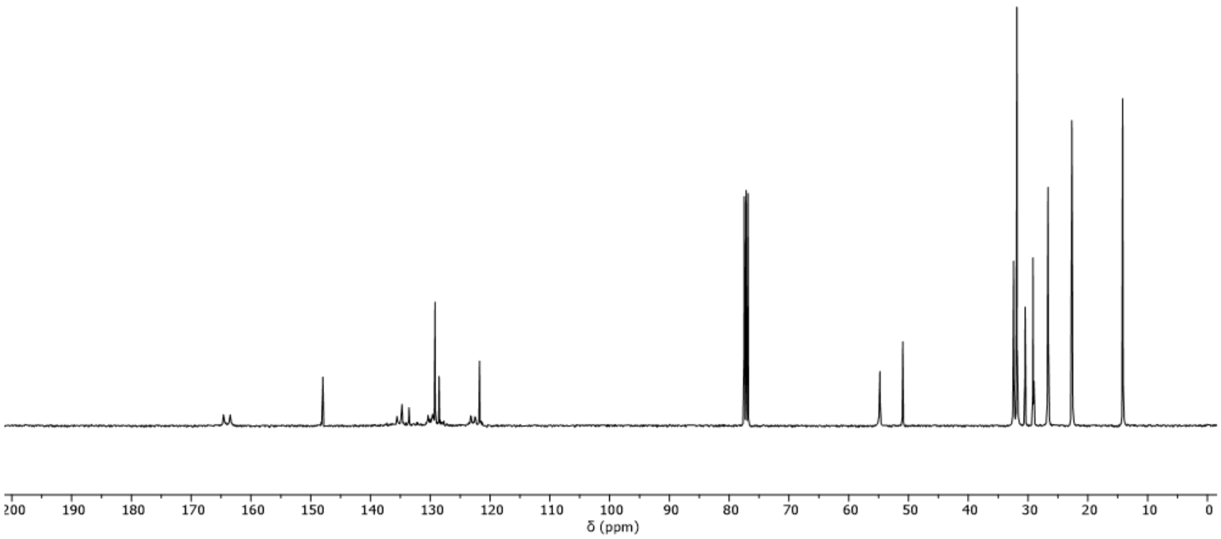

^{13}C NMR spectrum of acyclic monomer **6** ($CDCl_3$, 298 K, 101 MHz).

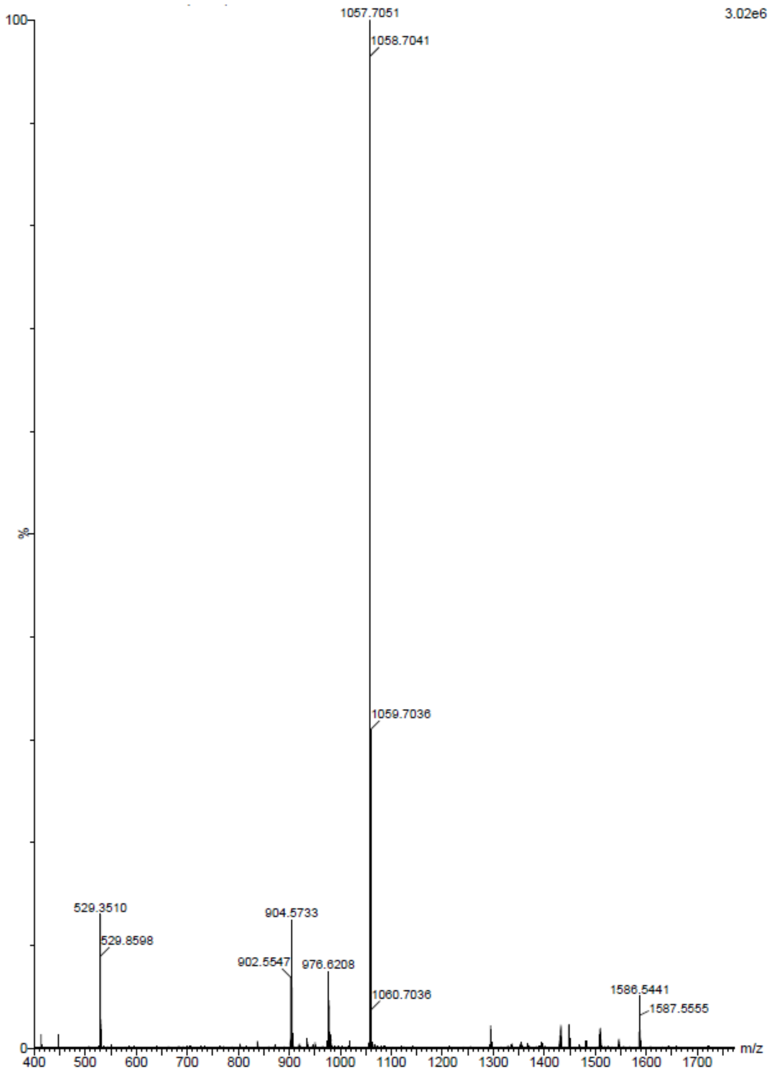

Observed ESI-MS data for monomer **6**.

3. Stereoisomer analysis

Isolation of the diastereoisomers of macrocycle 1 by HPLC

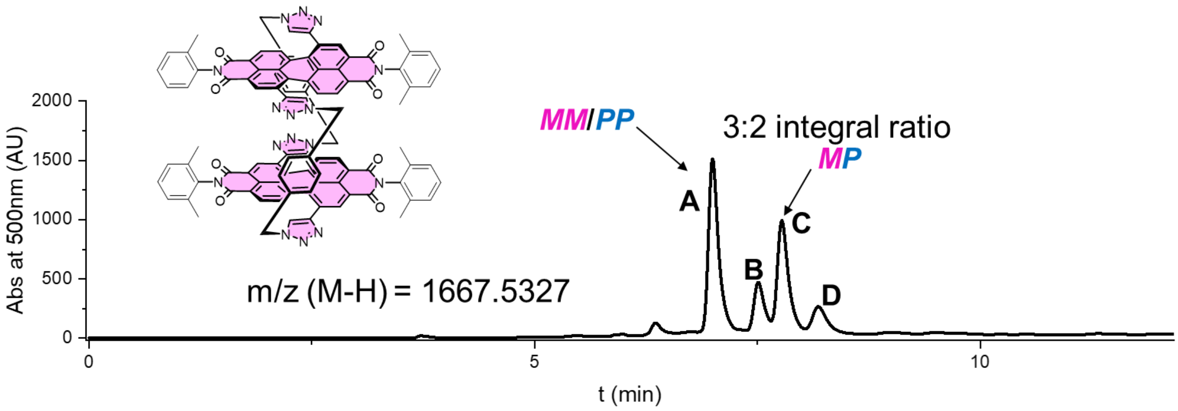

Figure S3-1: HPLC chromatogram of the crude reaction mixture (COSMOSIL Buckyprep 250 x 10 mm, eluted with a gradient going from 1:1 (v/v) DCM:n-hexane eluent to 7:3:1 (v/v/v) DCM:n-hexane:isopropanol) for the final macrocyclisation step in the synthesis of **1**, after filtration to remove insoluble side-products, likely extended PDI oligomers. Only peaks A and C contained the correct mass (m/z) for macrocycle **1** upon analysis by MALDI mass spectrometry. We propose that peaks B and D represent shorter, and thus more soluble, oligomeric side-products than those removed by the previous filtration. The 3:2 ratio of macrocycle products indicates there is a lower activation energy for forming **1-homo** compared to **1-meso** ($\Delta G^{\ddagger}$ = ~1 kJ mol^{-1}).

Resolution of macrocycle 1-homo enantiomers by chiral HPLC

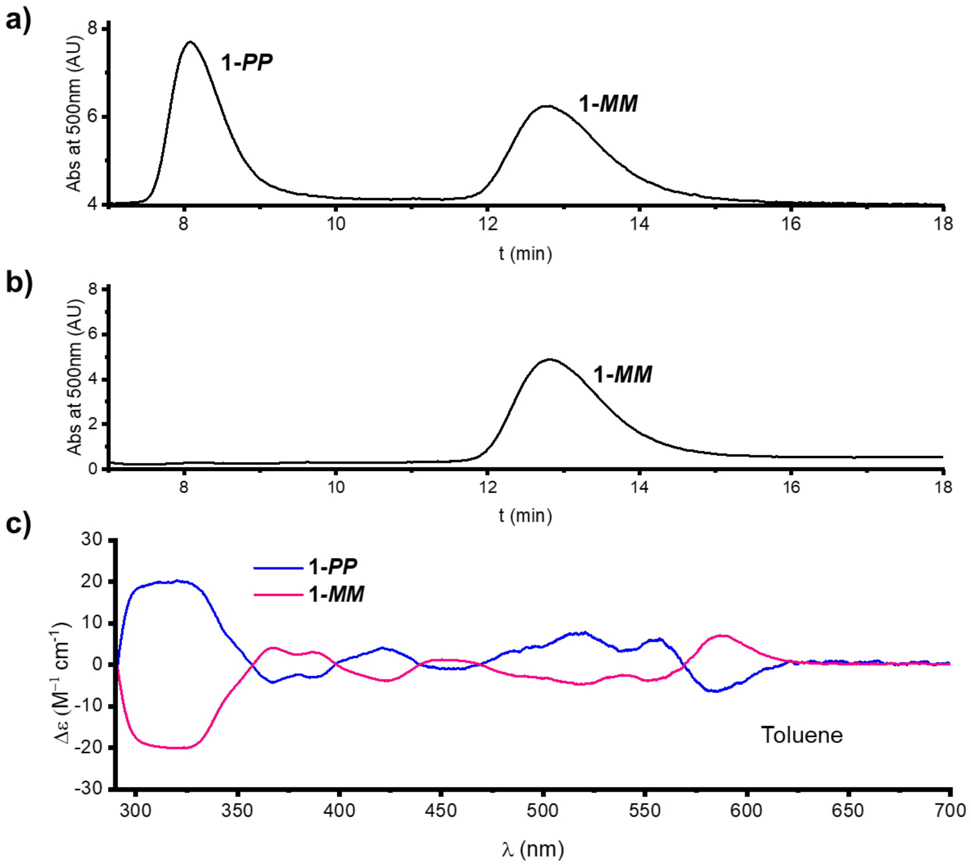

Figure S3-2: a) Chiral HPLC chromatogram of **1-homo** (Phenomenex i-Amylose-1, 250 x 10 mm, 7:3 (v/v) DCM:n-hexane eluent). b) Chiral HPLC chromatogram of the pure enantiomer **1-*MM*** that was heated at 180 °C for 24 h in 1,2-dicholorbenzene. After heating, the solvent was removed and the sample was re-dissolved in DCM and reinjected onto the chiral HPLC column and eluted with 7:3 (v/v) DCM:n-hexane. No formation of the opposite enantiomer **1-*PP*** can be detected in this chromatogram, proving that macrocycle **1-homo** is chirally locked at temperatures at least as high as 180 °C. c) CD spectra of the enantiomers **1-*MM*** and **1-*PP*** (toluene, 10μM).

Analysis of macrocycle 1-meso

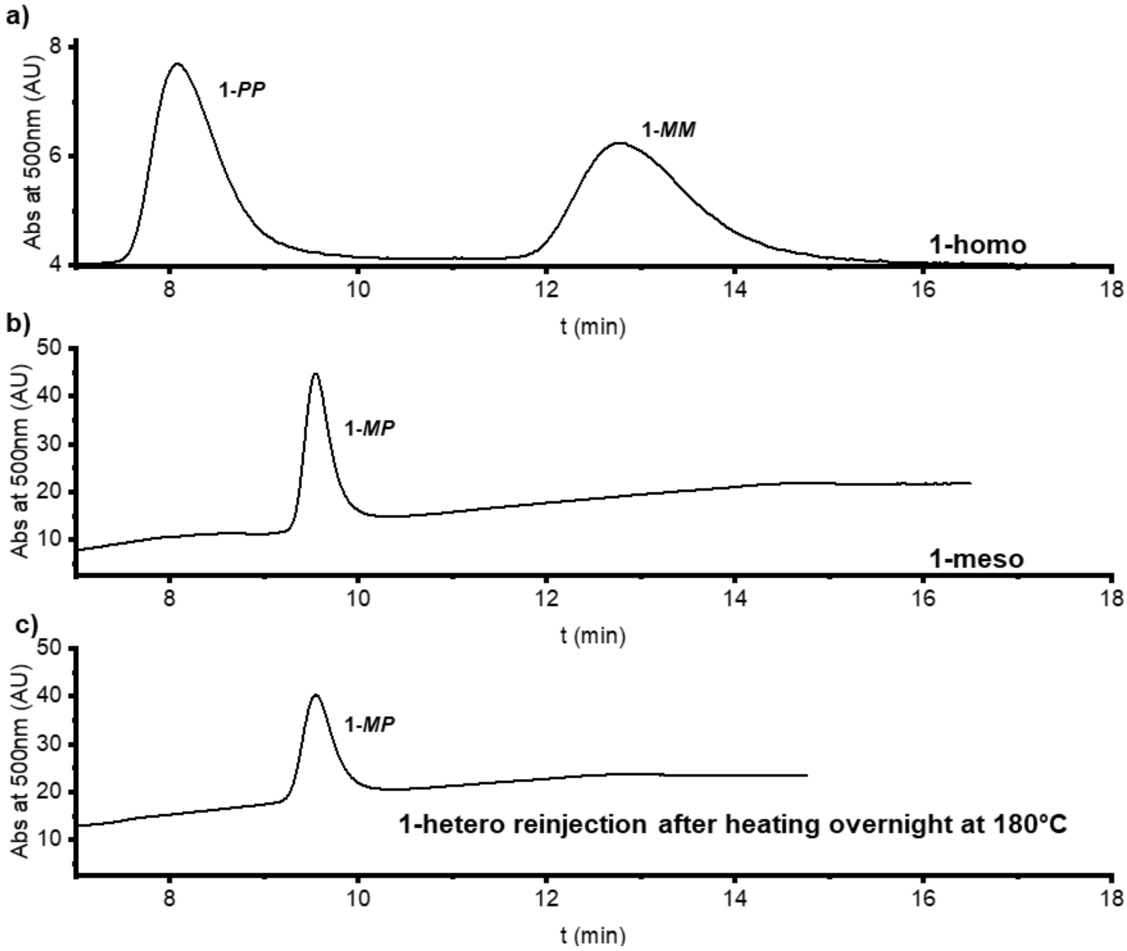

Figure S3-3: Chiral HPLC chromatograms (Phenomenex i-Amylose-1, 250 x 10 mm, 7:3 (v/v) DCM:n-hexane eluent) of a) **1-homo**, b) **1-meso**, and c) a sample of **1-meso** that was heated at 180°C for 24 h in 1,2-dichlorobenzene. After heating, the solvent was removed, and the sample was re-dissolved in DCM and reinjected onto the chiral HPLC column.

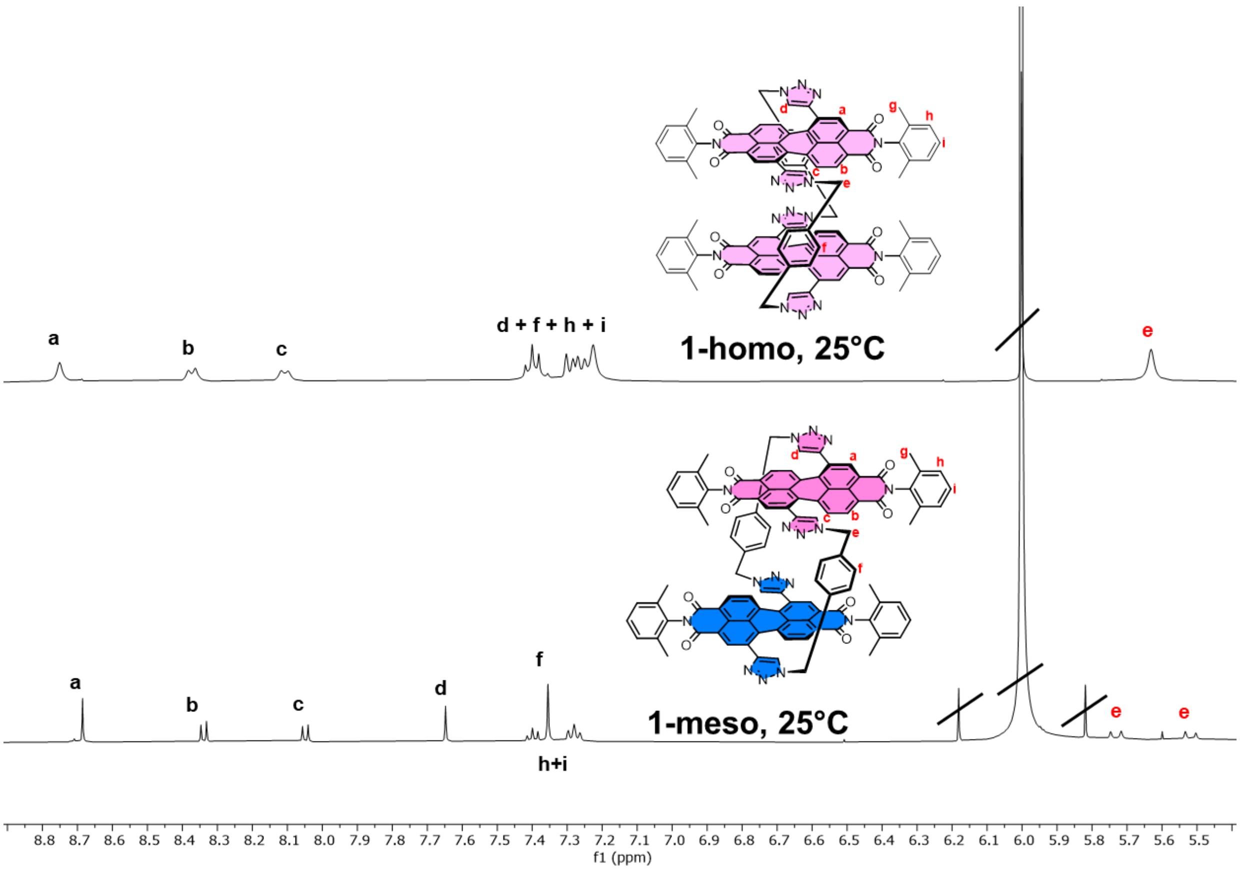

Figure S3-4: ^{1}H NMR spectra (TCE-d_2, 25°C, 400 MHz) of macrocycle **1-homo** (top) and **1-meso** (bottom).

4. Conformational switching studies for 1-homo

UV-vis absorption and circular dichroism studies

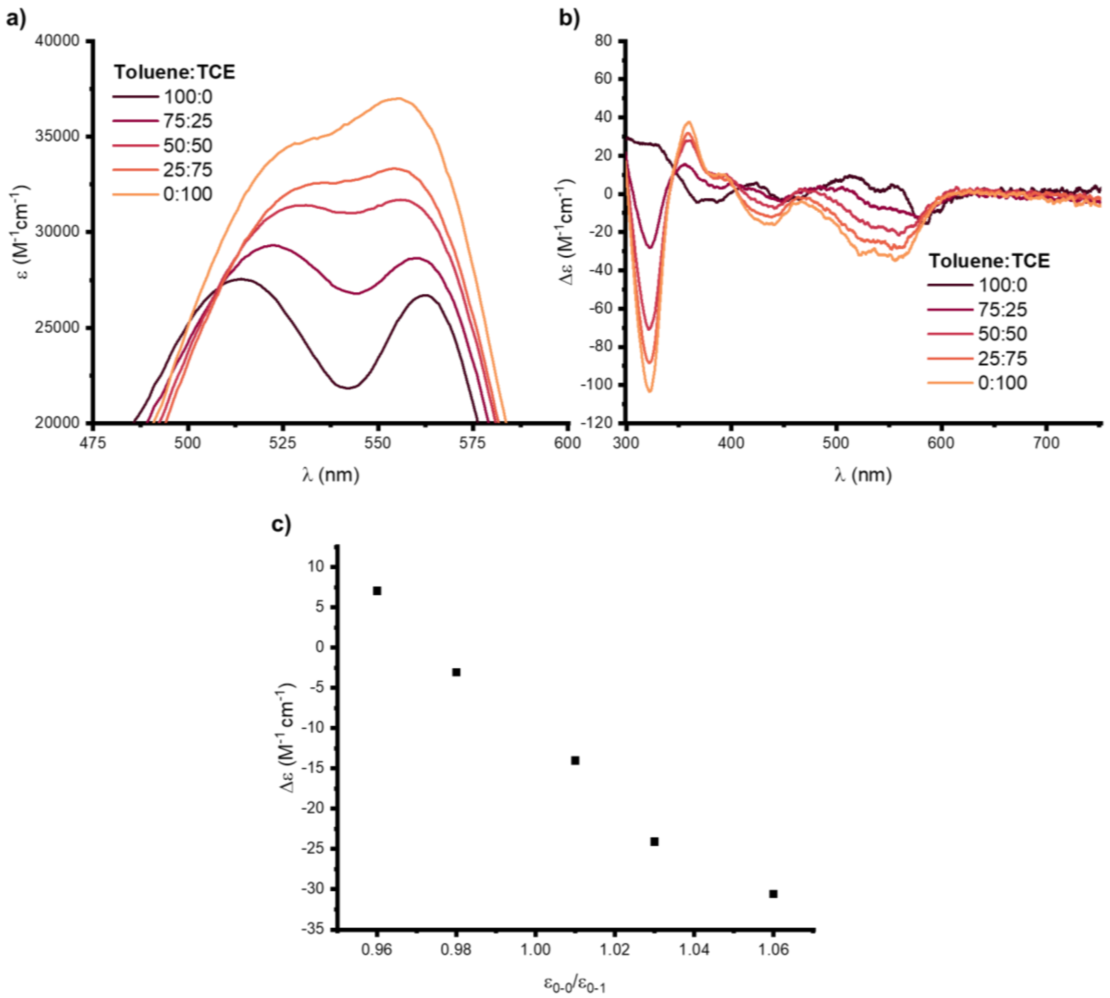

Figure S4-1: a) UV-vis absorption spectrum of macrocycle **1-homo** in different toluene:TCE mixtures. b) CD spectra of the same enantiomer **1-*PP*** in different toluene:TCE mixtures. c) Relationship between circular dichroism at 526 nm and vibronic peak ratio $\varepsilon_{0\text{-}0}/\ \varepsilon_{0\text{-}1}$ for the $S_0 \rightarrow S_1$ transition determined from the UV-vis absorption spectra in (b).

NMR studies

The solvent-dependent conformational change in **1-homo** was also studied by ^{1}H NMR spectroscopy (Figure S4-2). Considerable upfield shifts of the aromatic protons are observed in **1-homo** when toluene-d_8 is added to a solution in TCE-d_2, in particular proton H_c ($\Delta\delta$ = 1.5 ppm), which is consistent with toluene promoting intramolecular π–π interactions between the PDI units.[7]

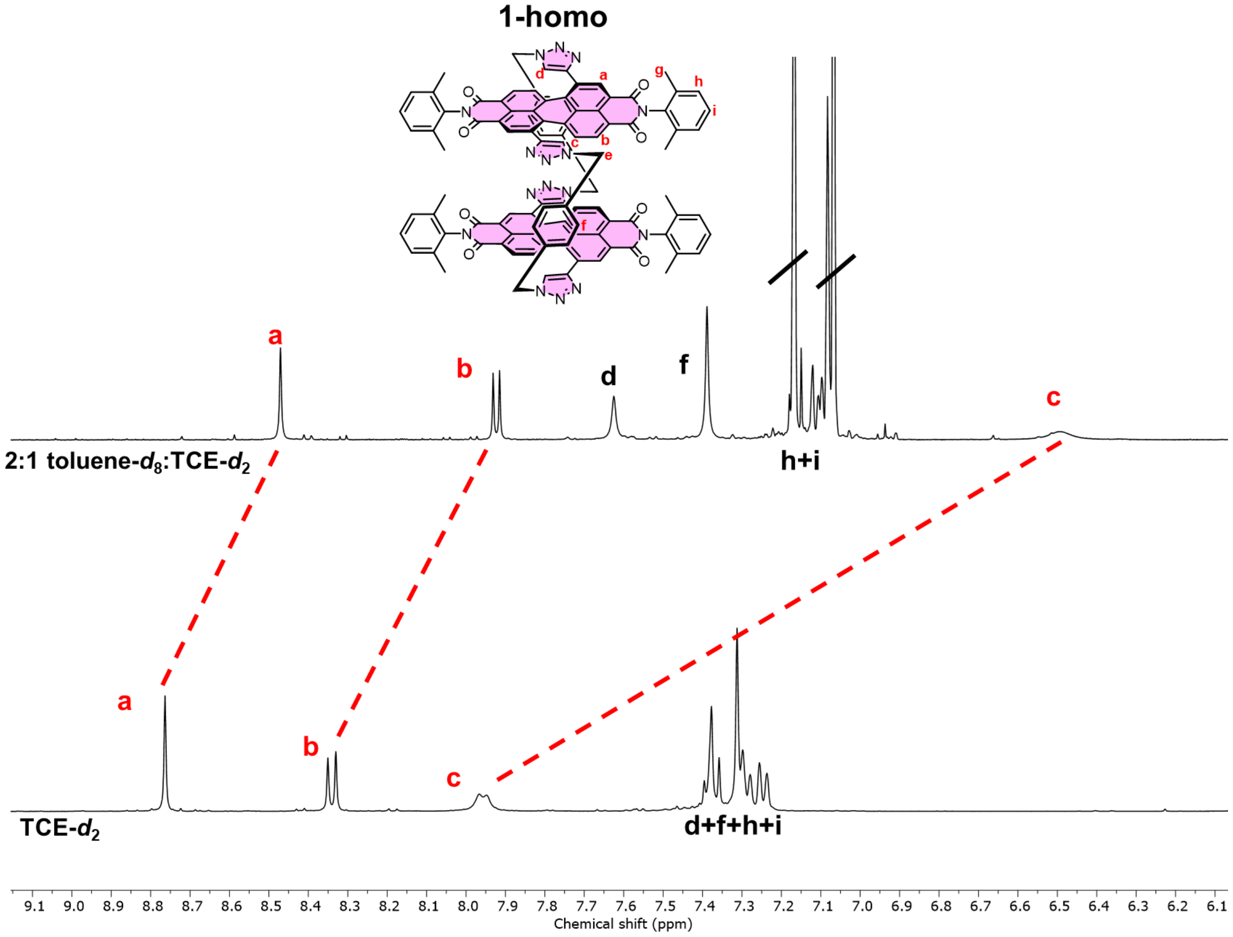

Figure S4-2: ^{1}H NMR spectra (70°C, 400 MHz, each referenced to the same internal standard, poly(dimethylsiloxane)) of macrocycle **1-homo** in 2:1 toluene-d_8:TCE-d_2 (top) and TCE-d_2 (bottom).

Fluorescence studies

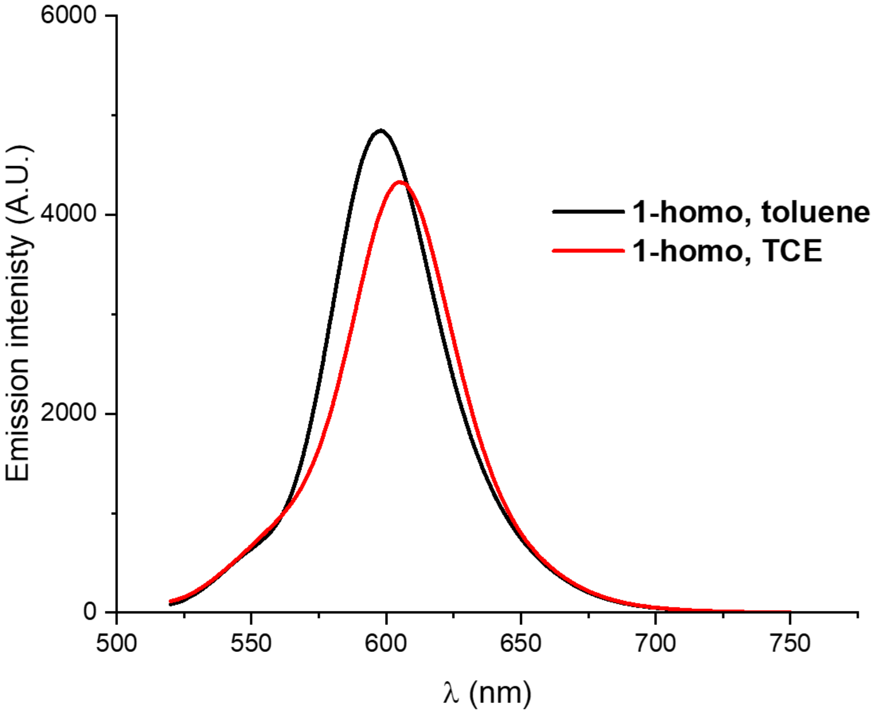

Figure S4-3: Fluorescence emission spectra (10 µM, λ_{ex} = 500 nm) of macrocycle **1-homo** in toluene (black trace) and TCE (red trace).

5. Conformational switching studies for 1-meso

NMR studies

The ^{1}H NMR spectra of **1-meso** and **1-homo** are very similar in TCE-d_2. However, the addition of toluene-d_8 has an opposite effect on the ^{1}H NMR spectrum. For **1-homo**, the PDI signals H_{a-c} experience a significant upfield shift relative to the spectrum in TCE-d_2 ($\Delta\delta$ = 0.4–1.5 ppm, Figure S5-1a). In line with UV-vis and CD spectroscopic data, this is evidence for co-facial intramolecular PDI-PDI stacking in **1-homo**. In contrast, for **1-meso**, the PDI signals H_{a-c} shift moderately downfield ($\Delta\delta$ = 0.1-0.3 ppm) upon the addition of toluene (Figure S5-1b), indicating that a distinct conformation is now adopted.

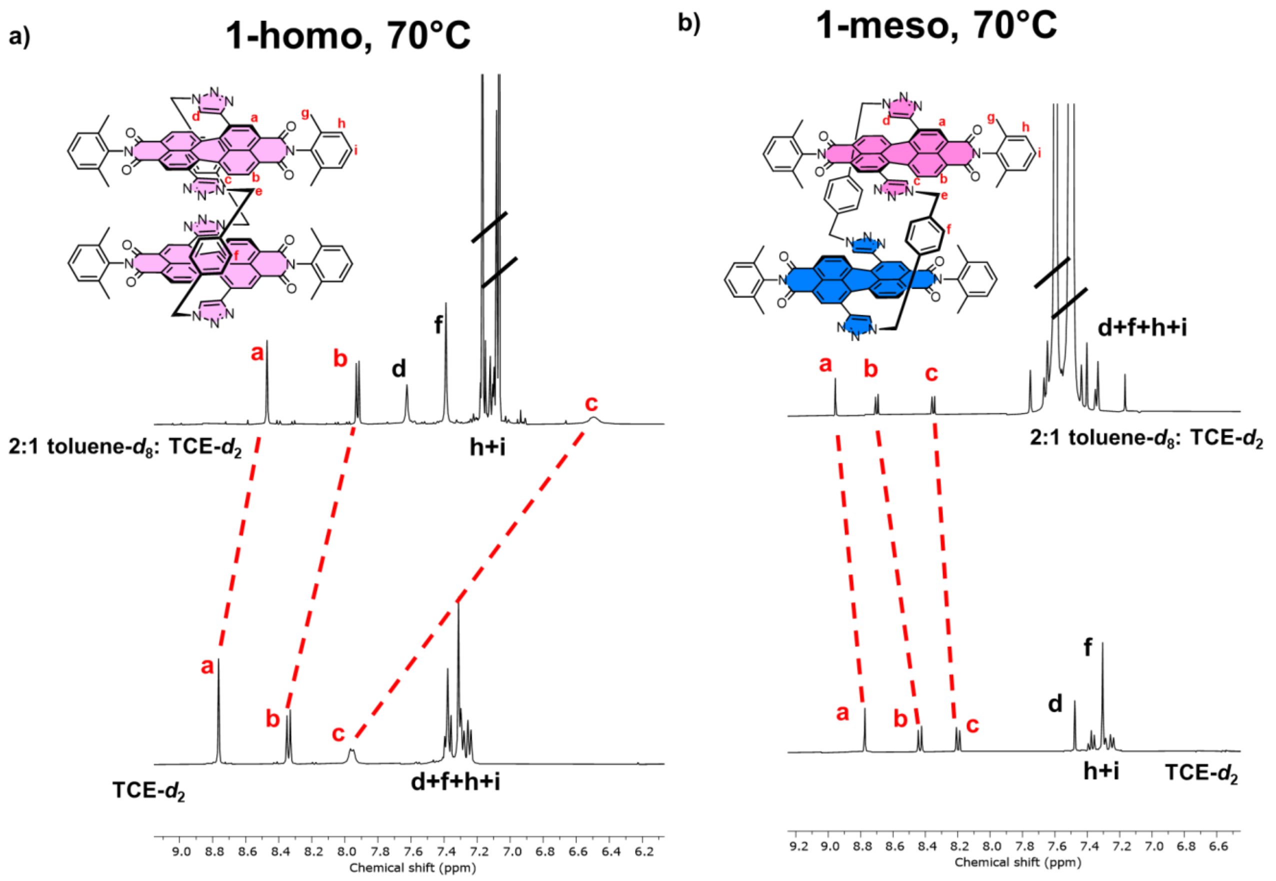

Figure S5-1: Stacked ^{1}H NMR spectra (75°C, 400 MHz) of macrocycle (a) **1-homo** and (b) **1-meso** in TCE-d_2 and 2:1 toluene-d_8:TCE-d_2. The spectra are aligned using an internal reference standard, poly(dimethylsiloxane), added to each sample.

Fluorescence studies

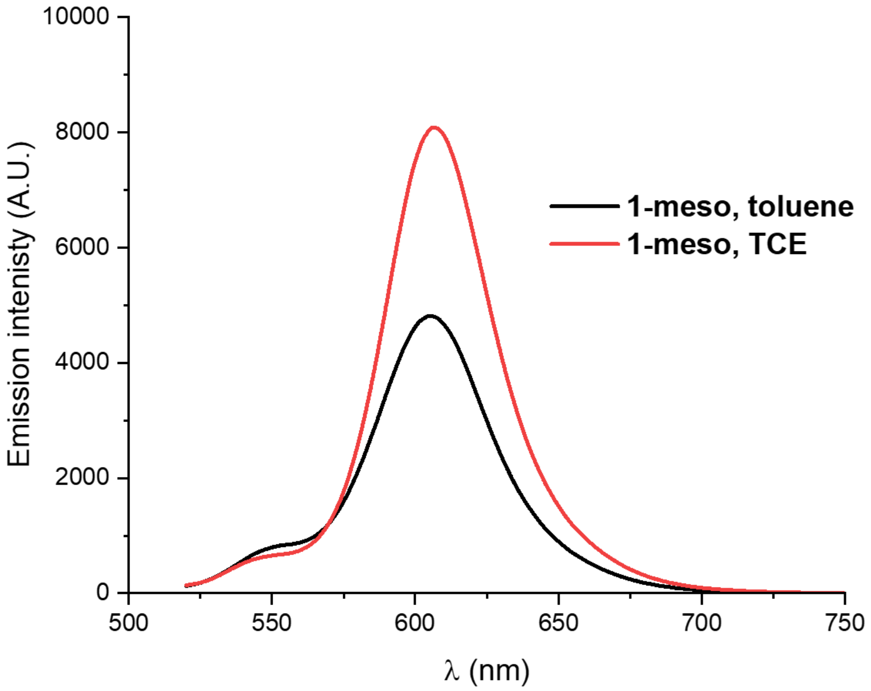

Figure S5-2: Fluorescence emission spectra (10 μM, λ_{ex} = 500 nm) of macrocycle **1-meso** in toluene (black trace) and TCE (red trace).

Table S5-1: Quantum yields for compounds **1-homo** and **1-meso** in toluene and TCE.

Compound	Solvent	Quantum Yield
**1-homo**	Toluene	0.40
	TCE	0.60
**1-meso**	Toluene	0.2
	TCE	0.4

6. (Multiphoton) CPL spectroscopy

Multiphoton Spectroscopy

The MP excitation process for macrocycle **1-homo** was shown to be a two-photon event at 760 nm as the laser power dependence of the two-photon induced emission at this excitation wavelength has a slope of 2 on a logarithmic scale .[8, 9]

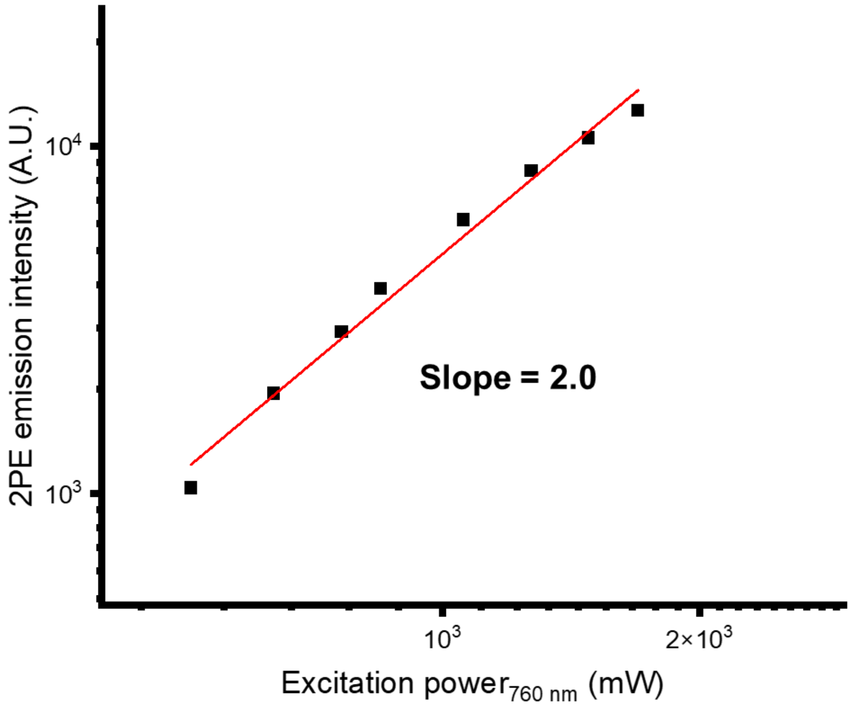

Figure S6-1: Excitation power dependence of the two-photon excitation (2PE) luminescence emission intensity (λ_{ex} = 760 nm), slope = 2.0 ± 0.1.

The two-photon absorption cross section (σ_2) of macrocycle **1-homo** were calculated according to equation S1 following established procedures. [10, 11]

$$\frac{\sigma_{2,S}\,\phi_S}{\sigma_{2,R}\,\phi_R} = \frac{C_R\, n_s F_S(\lambda)}{C_S\, n_R F_R(\lambda)} \qquad \text{(S1)}$$

Where *S* indicates the sample, *R* indicates the reference compound, ϕ is the total emission quantum yield, C is the concentration, n is the refractive index of the solvent, and $F_S(\lambda)$ and $F_R(\lambda)$ are the integrated photoluminescence spectra for the sample and reference respectively. The cross sections were calculated with respect to Rhodamine B in methanol. The two-photon excitation photoluminescence spectra used for **1-homo** in toluene was thus calculated as 388 GM (λ_{ex} = 760 nm), where 1 GM = 10^{-50} cm^4 s photon^{-1}.

Multiphoton CPL Spectroscopy

The CPL spectra below were measured in toluene and TCE for the enantiomers of **1-homo** using excitation at 760 nm, a wavelength which involves a two-photon absorption process as established above.

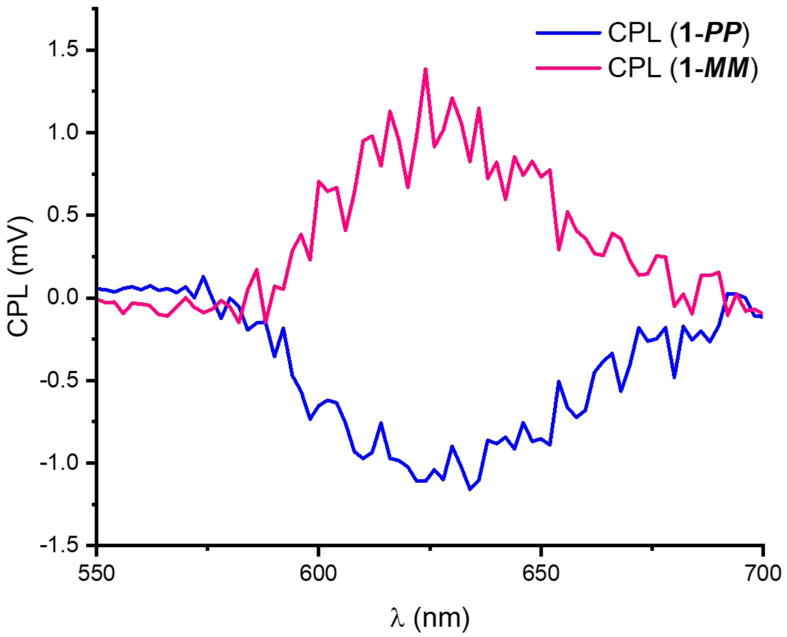

Figure S6-2: MP-CPL (λ_{ex} = 760 nm) spectra for **1-*MM*** and **1-*PP*** in toluene.

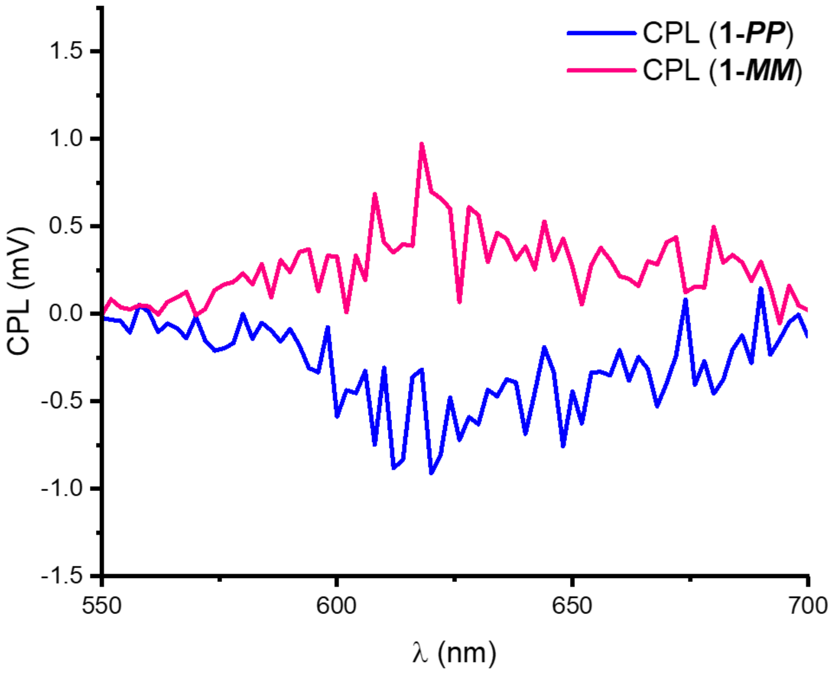

Figure S6-3: MP-CPL (λ_{ex} = 760 nm) spectra for **1-*MM*** and **1-*PP*** in TCE.

Single photon excitation CPL Spectroscopy

The CPL spectra were also measured in toluene and TCE using single photon excitation at 475 nm.

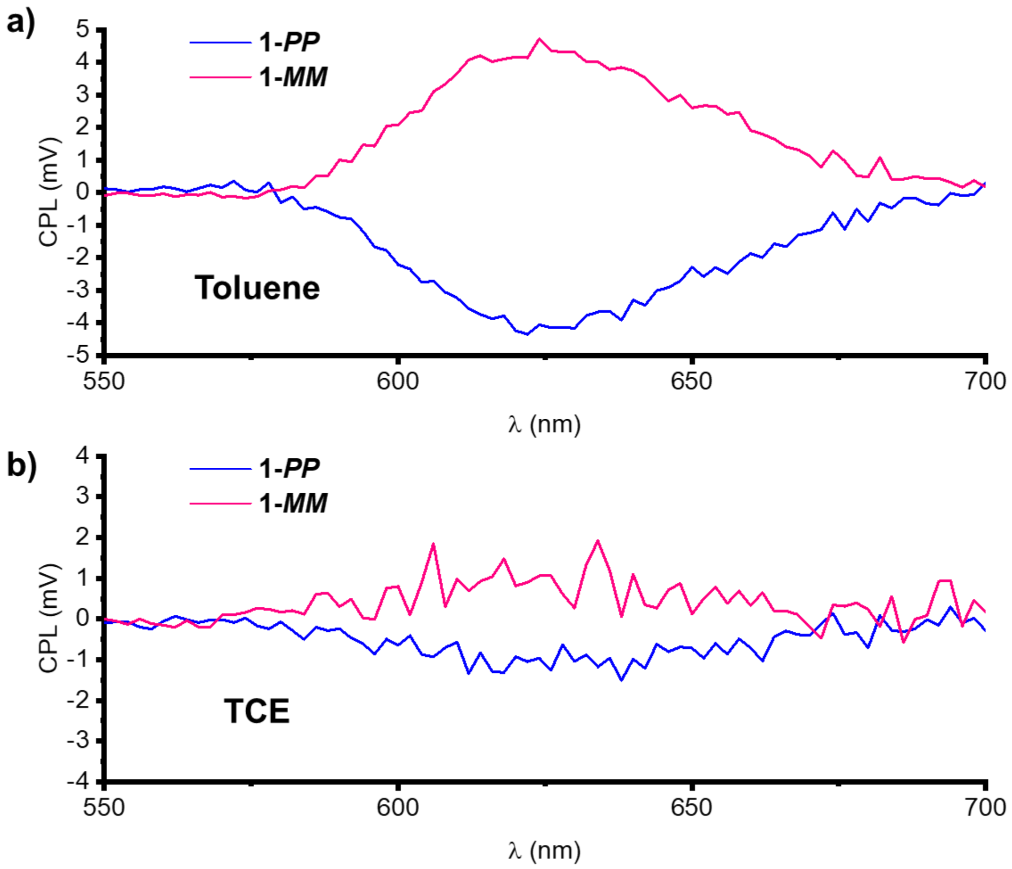

Figure S6-4: CPL spectra of the enantiomers of macrocycle **1-homo** (10 μM, λ_{ex} = 475 nm) in (a) toluene and (b) TCE.

7. Time-resolved infrared (TRIR) spectroscopy

FTIR

FTIR recorded for the bis-PDI macrocycle dimer **1-homo** and PDI monomer **6** in dry DCM and toluene (1mM) show four vibrational modes between 1500 cm^{-1} and 1800 cm^{-1} (Figure S7-1 and Table S1). Two ν(C=C) modes are associated with the aromatic rings around 1589.3 cm^{-1} and 1600.9 cm^{-1}. The positions of these peaks in toluene are slightly obscured by a toluene peak and water. A further two peaks associated with ν(C=O) stretches are observed between 1650 cm^{-1} and 1700 cm^{-1}. For the macrocycle dimer the peaks shift between DCM and toluene from 1656.5 cm^{-1} and 1695.4 cm^{-1} to 1652.5 cm^{-1} and 1695.1 cm^{-1}. The FTIR of the dimer in both solvents show an additional feature between 1700 cm^{-1} and 1750 cm^{-1} that is not present in the FTIR recorded for the monomer, potentially due to the greater conformational flexibility of the bis-PDI macrocycle in comparison to the monomer. In the monomer these peaks are located at 1656.1 cm^{-1} and 1694.9 cm^{-1} in DCM and 1660.2 cm^{-1} and 1696.6 cm^{-1} in toluene.

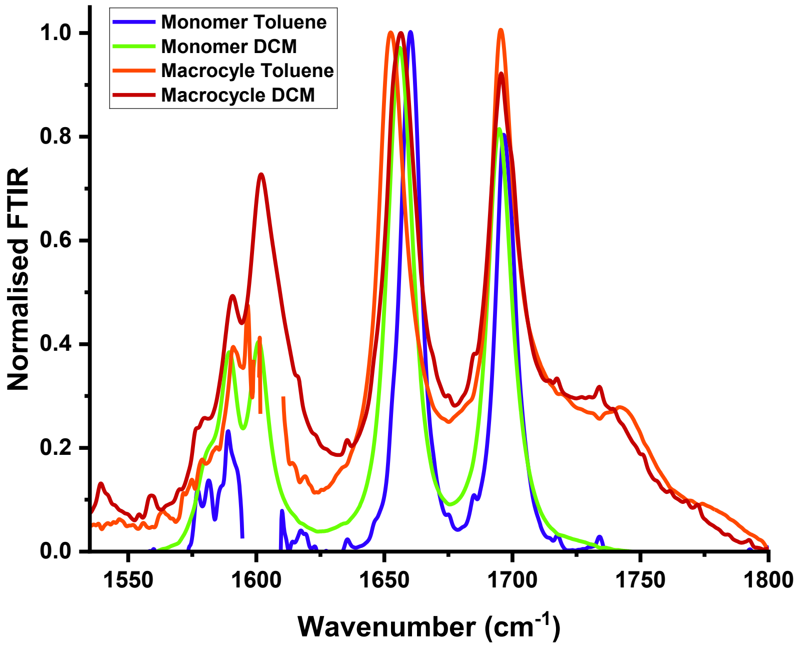

Figure S7-1: FTIR of the monomer and macrocycle in dry toluene and DCM (1mM).

Table S7-1. A list of vibrational modes of the monomer and macrocycle in dry toluene and DCM.

**Sample/Solvent**	**ν(C=C)/cm^{-1}**	**ν(C=O)/cm^{-1}**
Monomer/DCM	1589.3, 1600.9	1656.1, 1694.9
Monomer/Toluene	~1588.8	1660.2, 1696.6
Macrocycle/DCM	~1590.7, 1601.6	1656.5, 1695.4
Macrocycle/Toluene	~1591.0	1652.5, 1695.1

TRIR

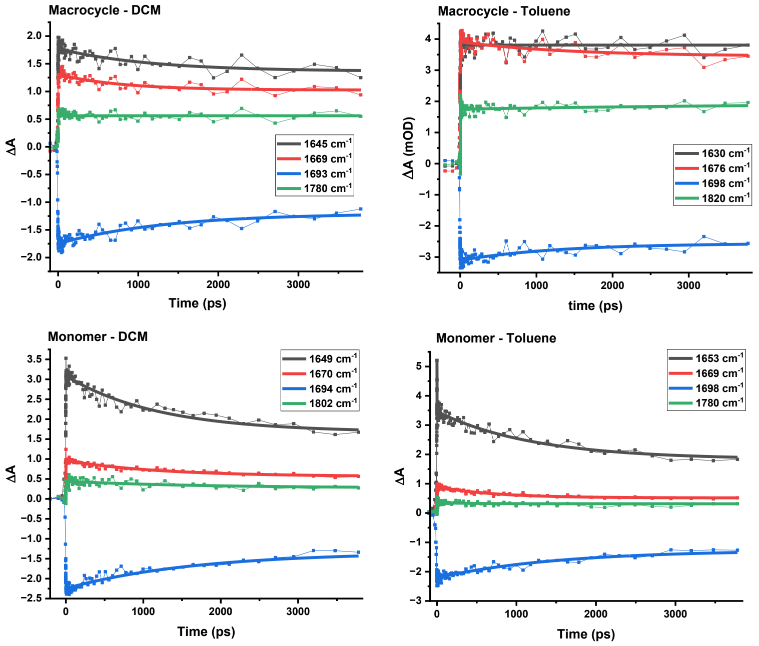

Figure S7-2: Time-resolved IR: Kinetic traces at selected frequencies, as stated.

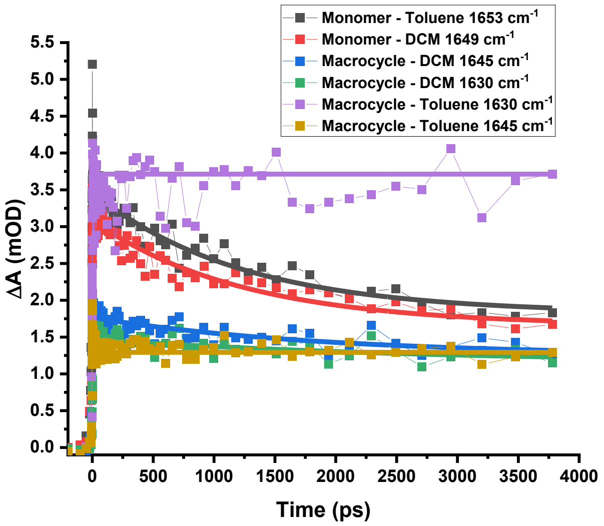

Figure S7-3: TRIR kinetics comparison of the transient features at 1649-1653 cm^{-1} and 1630 cm^{-1}.

Table S7-2: Kinetic parameters for 1649 cm^{-1} – 1654 cm^{-1} and 1630 cm^{-1} transient IR features for the monomer and macrocycle in toluene and DCM

**Macrocycle 1-homo**	**1649 - 1654 cm^{-1}**		**1630 cm^{-1}**
	τ_1 / ps	τ_2 / ps	
**DCM**	0.3	1970 ± 300	2700 ± 660
**Toluene**	6.45 ± 1.73	Infinity (on the timescale of experiment)	Infinity

8. Density Functional Theory Calculations

Conformer searches for macrocycle **1-homo** were performed using the CREST[12] code and GFN2-xTB tight-binding method.[13] The lowest energy conformers (both ***PP*** and ***MM*** enantiomers) were subsequently reoptimized by density functional theory using the B97-3c composite scheme by Brandenburg and co-workers.[14] Solvation effects (toluene or 1,1,2,2-tetrachloroethane [TCE]) in the DFT calculations were described using the COSMO implicit solvation model.[15] The two lowest energy conformations after DFT reoptimisation are conformers **A** and **B**, shown in Figure S8-1a and Figure S8-1b respectively and provided as .xyz files in the “DFT structures” folder. In the gas phase, the conformation in which the PDI’s bay substituents are directed away from the macrocycle cavity (i.e., **1-*PP*** in conformer **A**, Figure S8-1a) is 21 kJ mol^{-1} lower in energy than the conformation in which they point into the cavity (**1-*M**M**** in conformer **B**, Figure S8-1b).

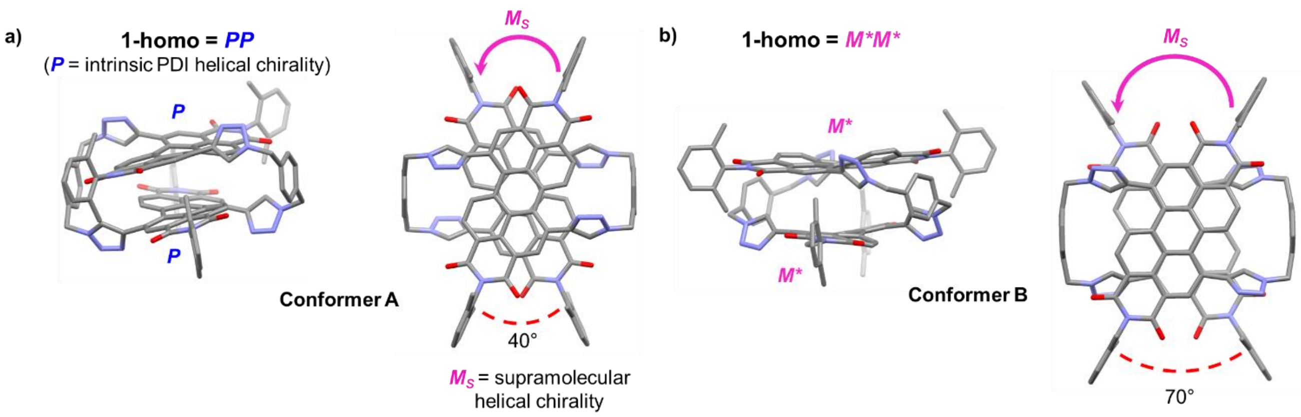

Figure S8-1: Structures of the two lowest energy conformers **A (a)** and **B (b)** found in the conformer search (only the ***PP*** **(*M*s)** enantiomer is shown).

Vertical excitation and circular dichroism spectra of the lowest energy DFT optimised conformer (**A**) of **1-homo** (Figure S8-1a) were calculated for both ***PP*** and ***MM*** enantiomers by TD-DFT using the ωB97x functional[16] and the def2-SVP[17] basis-set in toluene.

Table S8-1: TD-ωB97x/def2-SVP predicted circular dichroism spectra for the ***MM*** and ***PP*** enantiomers of the lowest energy conformer of **1-homo** in toluene (conformer **A**).

	**Excitation energy / eV (wavelength / nm)**	**Rotary strength / 10^{-40} erg cm^3**	
		MM	***PP***
1	2.42 (513)	542.13	-542.66
2	2.54 (488)	-768.47	769.14
3	3.21 (386)	2.35	-2.33
4	3.24 (383)	175.57	-175.71
5	3.69 (336)	0.00	0.00
6	3.71 (334)	-104.08	104.10

9. References

1. T. R. Chan, R. Hilgraf, K. B. Sharpless and V. V. Fokin, *Organic Letters*, 2004, **6**, 2853-2855.
2. L. E. MacKenzie, L.-O. Pålsson, D. Parker, A. Beeby and R. Pal, *Nature Communications*, 2020, **11**, 1676.
3. P. Stachelek, L. MacKenzie, D. Parker and R. Pal, *Nature Communications*, 2022, **13**, 553.
4. M. Franceschin, A. Alvino, G. Ortaggi and A. Bianco, *Tetrahedron Letters*, 2004, **45**, 9015-9020.
5. M. Queste, C. Cadiou, B. Pagoaga, L. Giraudet and N. Hoffmann, *New Journal of Chemistry*, 2010, **34**, 2537-2545.
6. E. J. O'Neil, K. M. DiVittorio and B. D. Smith, *Organic Letters*, 2007, **9**, 199-202.
7. S. E. Penty, M. A. Zwijnenburg, G. R. F. Orton, P. Stachelek, R. Pal, Y. Xie, S. L. Griffin and T. A. Barendt, *Journal of the American Chemical Society*, 2022, **144**, 12290-12298.
8. M. Göppert-Mayer, *Annalen der Physik*, 1931, **401**, 273-294.
9. W. Kaiser and C. G. B. Garrett, *Physical Review Letters*, 1961, **7**, 229-231.
10. C. Xu and W. W. Webb, *J. Opt. Soc. Am. B*, 1996, **13**, 481-491.
11. N. S. Makarov, M. Drobizhev and A. Rebane, *Optics Express*, 2008, **16**, 4029-4047.
12. P. Pracht, F. Bohle and S. Grimme, *Physical Chemistry Chemical Physics*, 2020, **22**, 7169-7192.
13. C. Bannwarth, S. Ehlert and S. Grimme, *Journal of Chemical Theory and Computation*, 2019, **15**, 1652-1671.
14. J. G. Brandenburg, C. Bannwarth, A. Hansen and S. Grimme, *The Journal of Chemical Physics*, 2018, **148**.
15. A. Klamt and G. Schüürmann, *Journal of the Chemical Society, Perkin Transactions 2*, 1993, DOI: 10.1039/P29930000799, 799-805.
16. J.-D. Chai and M. Head-Gordon, *The Journal of Chemical Physics*, 2008, **128**.
17. F. Weigend and R. Ahlrichs, *Physical Chemistry Chemical Physics*, 2005, **7**, 3297-3305.
